# Comparative functional analyses of the movement and coat proteins of grapevine Pinot gris virus, encoded by symptomatic and asymptomatic variants

**DOI:** 10.3389/fpls.2025.1659802

**Published:** 2025-09-25

**Authors:** Nikoletta Jaksa-Czotter, Emese Demián, Réka Sáray, Katalin Salánki, Éva Várallyay

**Affiliations:** ^1^ Genomics Research Group, Department of Plant Pathology, Institute of Plant Protection, Hungarian University of Agriculture and Life Sciences, Gödöllő, Hungary; ^2^ Department of Plant Pathology, Plant Protection Institute, Centre for Agricultural Research, Hungarian Research Network (HUN-REN), Budapest, Hungary

**Keywords:** RNA silencing suppressor, GPGV, GPGV-ORF3, GPGV-ORF2, necrosis

## Abstract

Using small RNA high-throughput sequencing (HTS), we previously demonstrated the widespread distribution of grapevine Pinot gris virus (GPGV) in Hungarian vineyards. This trichovirus has been associated with a disease known as grapevine leaf mottling and deformation (GLMD). However, since GPGV has been detected in both symptomatic and asymptomatic plants, its exact role in GLMD disease is not well-characterised. Studies addressing this question suggested that differences in the GPGV susceptibility of the grapevine cultivars and the presence of variants of the virus could affect symptom development. Being able to suppress various steps of the RNA interference-based defence reactions, the viral suppressor of RNAi (VSR), encoded by the ORF3 of GPGV, can also alter the symptom development. In the present study, we compared the VSR activity of the ORF3-encoded coat protein of symptomatic and asymptomatic GPGV variants and found that both possess VSR activity. Testing the VSR activity of the ORF2-encoded movement proteins from the two variants, using a GFP-based transient gene expression assay, we found that the GPGV-MP has weak systemic VSR activity. Moreover, we found that the transient expression of the MP variants induced necrosis in the infiltrated leaves, which was stronger in the case of the symptomatic variant. To functionally characterise the crucial sequence elements of MP responsible for this difference in the necrosis between symptomatic and asymptomatic variants, the necrosis-inducing activity of GPGV-MP encoded by different natural and recombinant variants was tested. Differences in the GPGV-MP necrosis-inducing activity suggested that, besides the previously described C/T polymorphism, different phosphorylation patterns of the GPGV-MP may contribute to symptom development.

## 1 Introduction

Grapevine Pinot gris virus (*Trichovirus pinovitis* – GPGV) is a member of the genus *Trichovirus* in the family *Betaflexiviridae* (Giampetruzzi et al., 2012). The virus was initially described in the grapevine cultivar Pinot Gris in Trentino, Italy, using small RNA (sRNA) high-throughput sequencing (HTS), which causes the grapevine leaf mottling and deformation disease (GLMD) (Giampetruzzi et al., 2012). Since its identification, GPGV has been found in several different grapevine cultivars in wine-producing countries across all five continents (EPPO: https://gd.eppo.int/taxon/GPGV00/distribution, updated October 17, 2024). Phylogenetic study based on historical isolates suggested that GPGV evolved in Asia (China/Japan) (Hily et al., 2020, 2021; Tokhmechi et al., 2021; Ben Mansour et al., 2024). GPGV likely entered Europe through Germany and eventually spread to the other continents (Hily et al., 2020; Ben Mansour et al., 2024). In the vineyard, it spreads by its slow-moving eriophyid mite vector, *Colomerus vitis* (Malagnini et al., 2016; Morán et al., 2018; Ulaşlı et al., 2024). However, the presence of GPGV in a wide range of various herbaceous and woody hosts such as *Silene latifolia*, *Chenopodium album, Ailanthus, Asclepias, Crataegus, Fraxinus, Rosa, Rubus*, and *Sambucus* species indicates the probable existence of additional insect vectors (Gualandri et al., 2017; Demian et al., 2022). In addition to its natural vector-based spread, GPGV is transmitted over long distances via infected propagation material (Diaz-Lara et al., 2021; Kaur et al., 2023).

GPGV has a positive-sense, single-stranded RNA genome of approximately 7259 nucleotides (nt), comprising three open reading frames (ORFs). ORF1 encodes replicase-associated proteins, methyltransferase, helicase, and RNA-dependent RNA polymerase (RdRp). ORF2 encodes the putative movement protein (MP), while ORF3 encodes the coat protein (CP) (Giampetruzzi et al., 2012).

Although GPGV has been directly associated with GLMD disease, its exact role and the reason for the differences in the severity of symptoms are still not fully understood. GPGV can be present in both symptomatic and asymptomatic grapevines, and although there are several hypotheses, the relationship between the presence of the virus and the appearance of symptoms is not yet completely clarified. Symptom development associated with GPGV can be explained by the genetic variance of the virus and the host. According to one of the earliest interpretations, the genetic diversity of GPGV strains, particularly, but not solely, the presence of a single-nucleotide (C/T) polymorphism at the 3’ end of the MP gene, can be the determining factor in the disease severity (Saldarelli et al., 2015). The differences at this position result in an early stop codon and a six-amino-acid shorter movement protein in the case of the symptomatic variants. Investigations of sequence variations in a large number of separately clustering GPGV (symptomatic and asymptomatic GPGV variants) showed variation at this position (Bertazzon et al., 2017; Marra et al., 2019; Tarquini et al., 2019a). Phylogenetic analysis of the MP/CP coding region of GPGV isolates showed that they segregate into three different clusters. In these detailed studies, several other SNP differences between the symptomatic and asymptomatic variants have been identified, and this is consequently why the original proposal about the role of the SNP at the end of the MP in the symptom development has been questioned (Bertazzon et al., 2017; Karki et al., 2025). Subsequently, the critical role of the 3’ end of the MP gene in the development of GLMD symptoms was experimentally investigated by Tarquini et al. (2019a). Based on the symptoms observed on the vines, they sequenced 9 different variants [symptomatic strains: fvg-ls9, fvg-ls12, and fvg-ls14 (they named them virulent); asymptomatic strains: fvg-ls1, fvg-ls6, fvg-ls8, fvg-ls13, fvg-ls15, and fvg-ls17 (they named them latent)]. Detailed sequence analyses of these (sometimes partial) clones suggested 39 SNPs, which separated isolates of the latent and virulent clades. Among them, eight are responsible for amino acid changes in the MP, including the originally described C/T polymorphism. A chimaera (containing the 253–370 amino acids of a latent fvg-ls15 in the virulent fvg-ls12 isolate) behaved as its asymptomatic fvg-ls15 parent, emphasising the key role of the 3’ end of the MP gene in symptom formation (Tarquini et al., 2019a, 2021a). Recently, several strains with ambiguous behaviour have been sequenced. A new GPGV variant has been sequenced in Canada (GPGV-SY), having a long MP (because of the C/T SNP), clustering with the asymptomatic clade, but causing severe symptoms. Experiments with its infectious clone further showed that it induced symptoms similar to the virulent variant. However, shortening its MP by mutating the C to T further increased the infectivity of the virus (Karki et al., 2025). In contrast, another asymptomatic variant (HU-27) clusters into the symptomatic clade, although it has a longer MP (Sáray et al., 2024). Besides the genetic variance, the manifestation of symptoms can also correlate with virus titre and can be altered by the tolerance/resistance of the infected grapevine cultivar, the boron deficiency, and the co-infection with other viruses (Bianchi et al., 2015; Bertazzon et al., 2017; Buoso et al., 2020).

RNA silencing, or RNAi, is a well-conserved sequence-specific RNA interference-mediated mechanism of gene regulation that also serves as an antiviral defence mechanism of the plants (Baulcombe, 2004). Antiviral RNA silencing is triggered by double-stranded RNA (dsRNA). These dsRNAs are recognised and processed by RNase III-type Dicer-like (DCL) enzymes into virus-derived 21–24 nucleotide (nt) long small interfering RNAs (vsiRNAs) that are incorporated into ARGONAUTE (AGO)-containing RNA-induced silencing complexes (RISCs), leading to sequence-specific degradation of target RNAs (Hamilton and Baulcombe, 1999; Ding et al., 2004; Carbonell et al., 2012). The RNA silencing signal can spread systemically in plants to trigger systemic silencing responses (Molnar et al., 2010). To counteract antiviral RNA silencing, most plant viruses evolved proteins that act as viral suppressors of RNA silencing (VSR) (Baulcombe, 2004; Ding and Voinnet, 2007; Burgyán, 2008). VSRs use different strategies to inhibit RNA silencing. RISC assembly can be inhibited by sequestering siRNAs by siRNA-binding proteins (Lakatos et al., 2006), while other VSRs impede the function of proteins involved in RNAi or mediate the degradation of AGO proteins (Csorba et al., 2015).

Plant defence reactions can play a key role in symptom severity. As they are affected by the efficiency of the VSRs, the severity of the symptoms can be directly connected to the presence and activity of VSRs.

During the molecular characterisation of GPGV, the investigation of the function of the GPGV-CP revealed its weak VSR activity (Tarquini et al., 2021b). In that report, only local VSR activity of the GPGV-CP, encoded by the ORF3 of the symptomatic fvg-ls12 variant, has been tested, leaving the question of the difference between CP-mediated VSR activities of the symptomatic and asymptomatic variants unanswered. In our previous survey, we identified and partially sequenced symptomatic and asymptomatic variants of GPGV in a Pinot gris cultivar in Hungary (Csókakő) (Demian et al., 2020).

In the present study, we tested and compared the local and systemic VSR activities of the CP and MP encoded by symptomatic and asymptomatic GPGV variants. We found that the GPGV-CP had only local VSR activity. In contrast, the GPGV-MP did not exhibit local, but showed weak systemic VSR activity. Moreover, transient expression of GPGV-MP induced local necrosis, which was more severe in the case of the symptomatic variant, suggesting that the variations in this protein may be an important determining factor of GPGV symptom development.

## 2 Materials and methods

### 2.1 Plants used in the study

Transgenic *Nicotiana benthamiana* plants stably expressing the GFP transgene (line 16c), wild-type (wt) *N. benthamiana*, *N. glutinosa*, and *N. tabacum* cv. Xanthi plants were grown under controlled conditions at 21°C, 16 h light/8 h dark. Infiltration assays were performed on expanded leaves of approximately 3–4-week-old plants. Plant material for local silencing experiments was harvested 4 days post-infiltration (dpi). For systemic silencing experiments, plants were observed from 4 to 21 dpi (every 3 to 4 days). The appearance of the leaf necrosis was observed and photographed at 5 dpi.

### 2.2 DNA constructs

ORF2 and ORF3 coding regions of HUCSK9s (MK953677.1), HUCSK8as (MK953676.1) (Demian et al., 2020), and HU-27 (PV208197) [ (Sáray et al., 2024) and this study] GPGV variants were cloned into the pJET1.2 vector (Thermo Fisher Scientific, USA). MPshort and MPmix GPGV variants were prepared using these clones and primers detailed in **Supplementary Tables 1**, **2**. The tested ORF coding sequences were amplified by CloneAmp HiFi PCR Premix (In-Fusion HD Cloning Kit, TaKaRa Bio, Inc., Shiga, Japan) according to the manufacturer’s recommendation and primers listed in **Supplementary Table 1**. The PCR products were purified (NucleoSpin Gel & PCR Clean-up Kit, Macherey-Nagel) and cloned into the BinHA vector (Nyikó et al., 2013) using the In-Fusion method (In-Fusion HD Cloning Kit, TaKaRa Bio, Inc., Shiga, Japan). The p19^CymRSV^ (from Cymbidium ringspot virus – CymRSV) (Várallyay and Havelda, 2013), 2b^CMV^ (cucumber mosaic virus – CMV) (Várallyay and Havelda, 2013), and P0^BWYV^ (beet western yellow virus - BWYV) (Csorba et al., 2010) VSR-expressing constructs used as positive controls were cloned as described above. A GFP-expressing binary vector was used as a silencing inducer in the infiltration assay (Johansen and Carrington, 2001). Confirmation of the clones was verified by Sanger sequencing of the constructs.

### 2.3 Transient expression assay – *Agrobacterium* co-infiltration

Each of the binary constructs was transformed into *Agrobacterium tumefaciens* strain C58C1 by conjugation (triparental mating method) in the presence of *Escherichia coli* pRK2013 helper plasmid. Transformed *A. tumefaciens* was grown at 30 °C overnight in YEB medium containing 50 µg/ml kanamycin, 5 µg/ml tetracycline, and 25 µg/ml rifampicin in 1M MES. The overnight culture was collected by centrifugation (4000 rpm for 10 min at room temperature), resuspended in 2.5 M MgCl_2_ and 1 M acetosyringone solution to a final OD_600_ = 1. In co-infiltration experiments, 0.4 volumes of pBinHaS-35S: GFP-expressing *Agrobacterium* were mixed with 0.6 volumes of individual VSR-expressing *Agrobacterium*. Young leaves of 4-week-old *N. benthamiana* plants were infiltrated with the mixture. The GFP fluorescence of infiltrated leaves (local silencing, 4 dpi) and whole plants (systemic silencing, 21 dpi) was observed visually under long-wave UV light (UVP Blak-Ray B-100APR, Analytik Jena, US) and photographed by using an Olympus PEN E-PL8 digital camera with a yellow filter (58 mm HTMC Gelb Mittel Y2 (8) Yellow Filter, Hama). For the molecular tests, 4 leaf samples were collected as a pool on the 3–4 dpi and processed further. Each experiment was repeated three times. Infiltrations in *N. glutinosa* and *N. tabacum* cv. Xanthi was repeated twice.

### 2.4 GFP fluorescence signal quantification

To quantify GFP fluorescence in the infiltrated patches of agroinoculated leaves of wt *N. benthamiana*, the green filter images of infiltrated leaves were uploaded into ImageJ software (Schneider et al., 2012). The fluorescence value of the same area of every infiltrated patch was calculated (6 patches/experiment) in three independent experiments and used for the statistical analysis.

### 2.5 Quantification of the systemic silencing

To assess the suppression of systemic silencing, 3-8 16c plants were infiltrated with each *Agrobacterium* mixture, and the spread of GFP silencing to the upper leaves was monitored over 3 weeks. GFP fluorescence was observed at 4, 7, 11, 14, and 21 dpi. The plants were photographed with an Olympus PEN E-PL8 camera using a yellow filter, and the number of all leaves and leaves showing systemic silencing were counted for each plant. GFP systemic silencing can be seen as the appearance of red tissue in newly growing leaves of the infiltrated plants. The systemic silencing efficiency was quantified by comparing the number of silenced leaves/all leaves of the plant in three independent experiments and used for the statistical analysis.

### 2.6 Quantification of the necrosis

To visualise and quantify cell death in the green, agroinfiltrated plant leaf tissues, we used a protocol based on the detection of necrotic area measurement (Laflamme et al., 2016; Pride et al., 2020). All of the images were captured using an Olympus PEN E-PL8 camera. ImageJ (Schneider et al., 2012) was used to quantify the necrotic area of the infiltrated patches. The necrotic area of 20 infiltrated patches/construct in three independent experiments was quantified and used for the statistical analysis.

### 2.7 RNA and protein extraction

Total nucleic acid was purified from the plant tissues (4 leaf samples as a pool). For RNA and protein analyses, 150–200 mg frozen leaves were ground in an ice-cold mortar to a fine powder in liquid nitrogen and homogenized in 355 μl extraction buffer (0.1 M glycine–NaOH, pH 9.0, 100 mM NaCl, 10 mM EDTA, 2% sodium dodecyl sulfate, and 1% sodium lauroyl sarcosinate) and divided into two aliquots. To one part (60 μl), one volume of 2× Laemmli buffer was added, boiled for five minutes, centrifuged at full speed for five minutes, and used as a protein sample. The remaining part was supplemented with 355 μl extraction buffer, and the total nucleic acids were purified using the phenol-chloroform method (White and Kaper, 1989). Nucleic acid concentration was quantified by the Nanodrop ND-1000 spectrophotometer (Thermo Fisher Scientific Inc., Waltham, MA, USA).

### 2.8 GFP protein quantification

*N. benthamiana* protein extracts (20 μl) were separated on a 12% SDS-polyacrylamide gel electrophoresis (SDS-PAGE). Electronically blotted onto PVDF Transfer Membrane (Amersham Hybond-P 0.45; Cytiva, USA) using a semi-dry blotting system (Bio-Rad, Trans-Blot^®^ Turbo™) and subjected to Western blot analysis. Membranes were blocked using 5% non-fat dry milk in phosphate-buffered saline (PBS) containing 0.05% Tween 20 (PBST) for 60 min at room temperature. Blots were incubated with anti-GFP-HRP conjugated antibody at a dilution of 1:10000 (Miltenyi Biotec, cat:130-091833) or anti-HA-peroxidase HRP conjugated antibody (Merck, cat:12013819001) at a dilution of 1:2000 in 1% non-fat dry milk in PBST for 1 h. At the same step for the normalisation control, membranes were incubated with BiP antibody (Agrisera AS09 481) for one hour at a dilution of 1:10000 in 1% dry milk (1× PBST) at room temperature. In the case of the Bip antibody, before the final step, the membrane was incubated with an HRP-conjugated goat anti-rabbit IgG secondary antibody (Agrisera, AS09 602) for 1 h at a dilution of 1:10000 in 1× PBST with agitation. Blots were washed three times for 5 min with PBST and finally developed using High Clarity Western ECL (Biorad) on ChemiDoc™ MP Imaging System (Biorad) in signal accumulation mode. The intensity of the signal was quantified using Image Lab 5.0 using the BIP signal for normalisation. The results of the quantification of three independent experiments were used for the statistical analysis.

### 2.9 GFP mRNA quantification

From the total nucleic acids, DNA was removed using DNase treatment. 5 μg of the total nucleic acid extracts were treated with TURBO DNA-free™ Kit (Invitrogen, Waltham, Massachusetts, USA) according to the manufacturer’s description. This purified, DNA-free RNA was used for cDNA synthesis with High-Capacity cDNA Reverse Transcription Kit (Applied Biosystems™, Waltham, Massachusetts, USA). Real-time PCR experiments were performed on a LightCycler^®^ 96 Instrument (Roche, Basel, Switzerland). RT-qPCR was carried out with 6 μl of 2x Power SYBR Green PCR Master Mix (Applied Biosystems™, Warrington, UK). Primer sequences for the quantification of GFP mRNA are listed in **Supplementary Table 1**. Every reaction was performed at 95°C for 10 min, 40 cycles of 95°C for 15 s, and 60°C for 60 s, followed by melting curve analysis from 65 to 95°C to check primer specificity. For the quantification, we used the △△Ct method, using the ubiquitin gene’s expression as a reference. For final quantification, the negative control with no template and three technical replicates were considered. The gained normalised expression values were used for the statistical analysis.

### 2.10 Statistical analysis

Statistical analysis was performed with GraphPad Prism 10. (Free version). The quantified data were analyzed using one-way ANOVA with p ≤ 0.05. For multiple comparisons, Tukey’s *post hoc* test was used. Statistical differences were marked using a compact letter display (CLD).

### 2.11 Northern blot detection of miRNAs

For small RNA Northern blot analyses, 5 μg of total nucleic acids were separated on denaturing 12% polyacrylamide gels containing 8 M urea and transferred to Nytran N membrane (Cytiva Whatman™) with semi-dry blotting. Membranes were chemically cross-linked. miR168 and miR159 were detected by miRNA-specific biotinylated LNA oligonucleotide with Chemiluminescent Nucleic Acid Detection Module Kit (ThermoFischer, USA) using our optimised protocol (Várallyay et al., 2008; Dalmadi et al., 2019). The expression was quantified on the band intensity measurement using ImageLab 5.0 (Bio-Rad).

### 2.12 Nucleic acid sequence and protein structure analysis

Multiple sequence alignment of the GPGV variants was performed using the Geneious Prime software (using the MUSCLE algorithm [5.1. version]). Evolutionary analysis was conducted using Geneious Tree Builder with the Jukes-Cantor model using the Neighbour-Joining method, of the same software, with 1000 bootstrap replicates to assess reliability.

For protein structure analysis, the Alphafold3 (Abramson et al., 2024) server was used. This program predicts a protein’s 3D structure from its amino acid sequence. ChimeraX 1.8 (https://www.rbvi.ucsf.edu/chimerax) (Pettersen et al., 2004; Meng et al., 2023) was used to visualise the protein structure and identify regions of structural variation.

For *in silico* phosphorylation site prediction, the free online phosphorylation site prediction software NetPhos 3.1 was used (https://services.healthtech.dtu.dk/services/NetPhos-3.1/), with its default settings. Probable phosphorylation sites were considered above a threshold value of 0.5, the phosphorylation of a given amino acid.

## 3 Results

### 3.1 Comparison of the CP and MP sequences encoded by the symptomatic and asymptomatic GPGV variants

**Figure 1:**
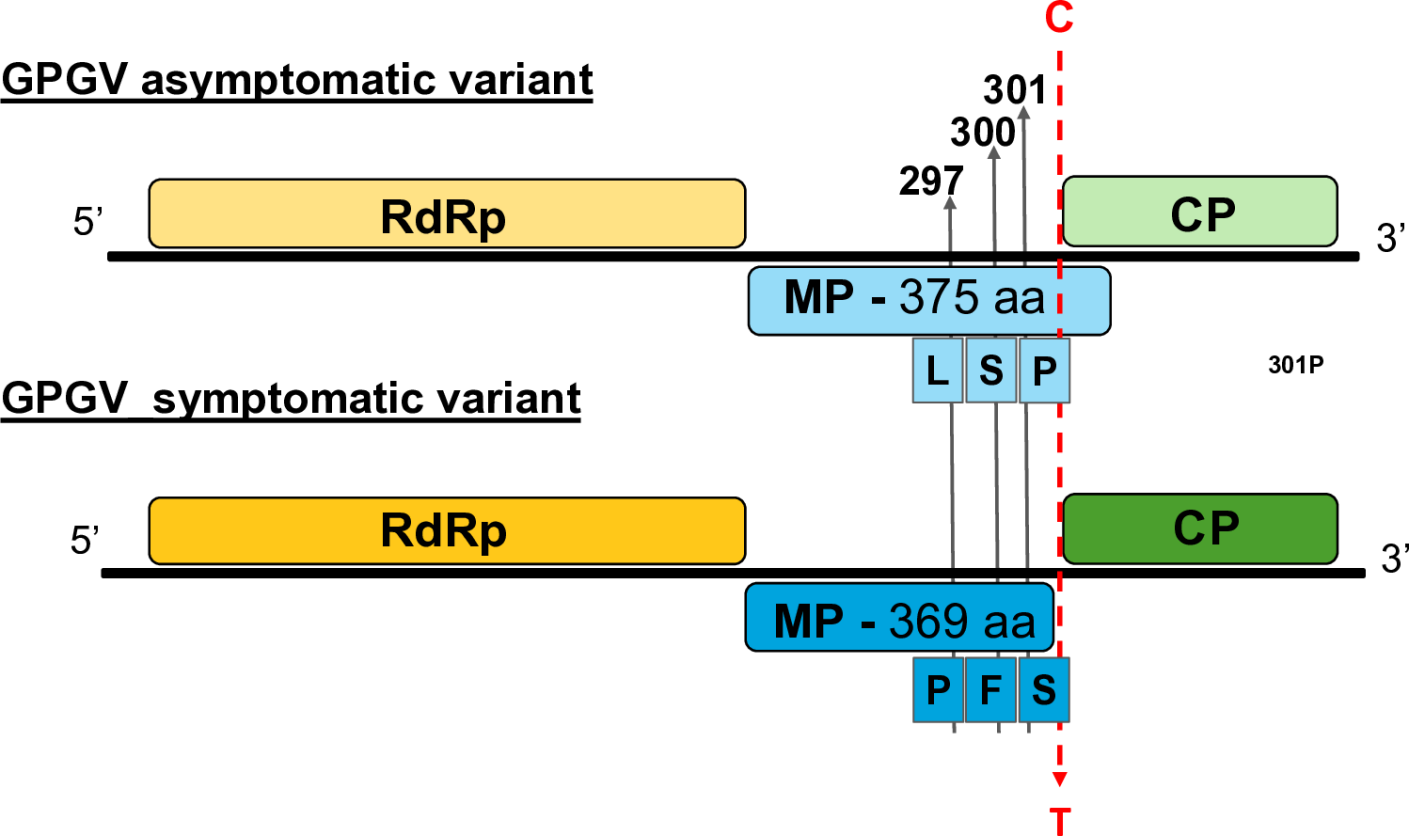
Key features of the symptomatic and asymptomatic GPGV variants. Schematic representation of the genome organisation of the symptomatic and asymptomatic GPGV variants with the position of some important polymorphic sites.

Investigating the possible causes of the symptomatic (s=virulent) and asymptomatic (as=latent) nature of GPGV, an SNP at the end of the MP (C/T polymorphism) was implicated. Due to this variation, the MPs in the symptomatic variants are six amino acids shorter (**Figure 1**). Phylogenetic analysis of the full GPGV genomes and partial sequences of the MP/CP coding region revealed the presence of several distinct clades. Numbering and nomenclature of these clades differ among authors, but they agree that the variants encoding symptomatic and asymptomatic strain cluster separately (**Supplementary Figure S1**). Phylogenetic analysis of the CP and MP alone resulted in similar clustering (**Supplementary Figures S2**, **S3**). The SNPs observed in the genomes led only to minor changes, resulting in very similar proteins, with higher than 94,5% and 98,6% identity for the CP and MP, respectively.

The CP of the virus appears to be very conservative; several distinct strains encode identical proteins (amino acid sequence of the sequenced part of the latent fvg-ls15 and virulent fvg-ls12 variants are identical), which is why the clustering on the amino acid level does not correlate with the symptoms (**Supplementary Figure S4**). Alignment of the CP amino acid sequences of symptomatic and asymptomatic GPGV strains revealed two positions that differ in the asymptomatic HUCSK8as and symptomatic HUCSK9s: amino acid 64 H/Y and 96 S/N. An alignment of the amino acid sequences of the MPs of these two strains and the well-characterised variants supported the presence of several amino acid differences between the two clades: 9 R/K, 277 F/L, 280 S/G, 297 L/P, 344 V/A, 355 E/K, 363 A/T, 366 V/A, and 370 Q/Stop, situated mainly at the carboxylic end of the protein (**Supplementary Figure S5**).

### 3.2 VSR activity of the GPGV-CP encoded by the symptomatic variant is stronger than the VSR activity of the asymptomatic variant

**Figure 2:**
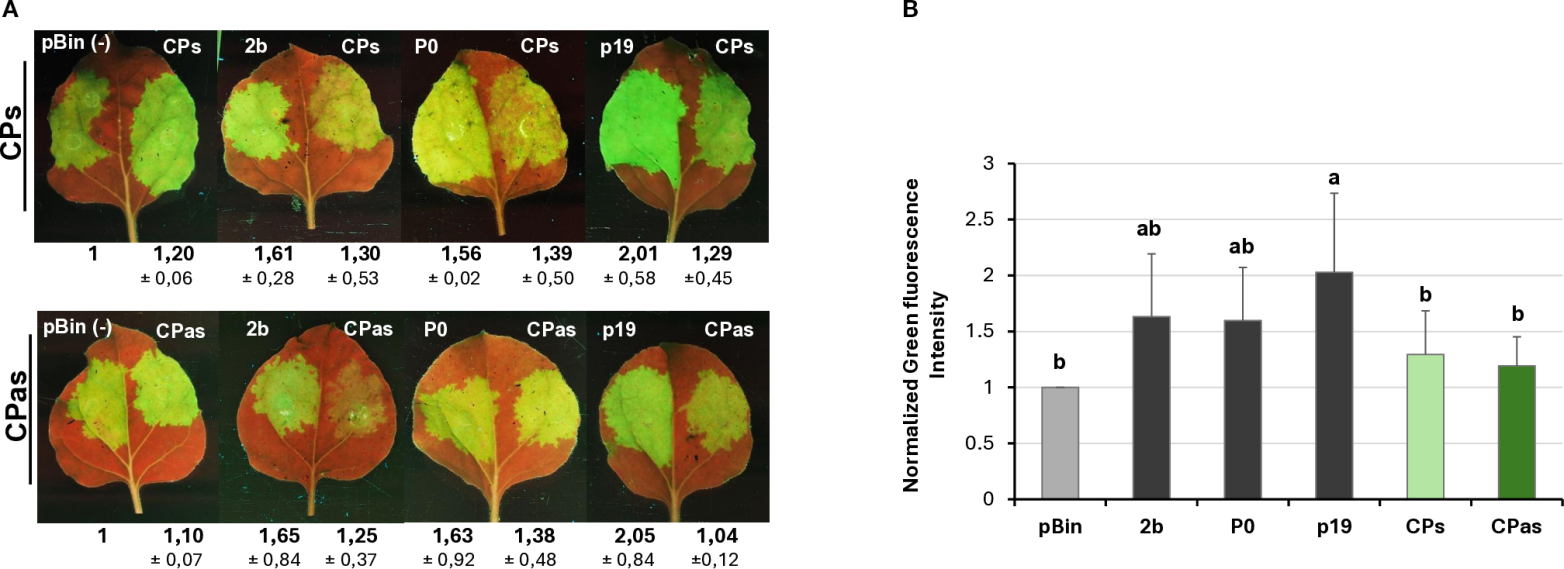
Transient silencing assay-based test of the local VSR activity of the GPGV-CP encoded by the symptomatic and asymptomatic variants. **(A)** Representative photos of leaves of wt *N. benthamiana* co-infiltrated with GPF and GPGV-CP (upper panel – CPs, lower panel – CPas). The empty vector (pBin) was used as a negative; p19^CymRSV^, 2b^CMV^, and P0^BWYV^ were used as positive controls. Photos were taken at 4 dpi under UV light. Each experiment was repeated three times. The fluorescence intensity of the infiltrated zones was quantified using ImageJ software. Bold numbers show the average fluorescence intensity of all of the infiltrated leaves of all three experiments, and ± indicates standard deviation. **(B)** Column diagram of the summarised result of the fluorescence intensity of the three independent experiments. The error bars indicate a standard deviation. Letters indicated significant difference at the 0.05 level according to one-way ANOVA and Tukey HSD test.

**Figure 3:**
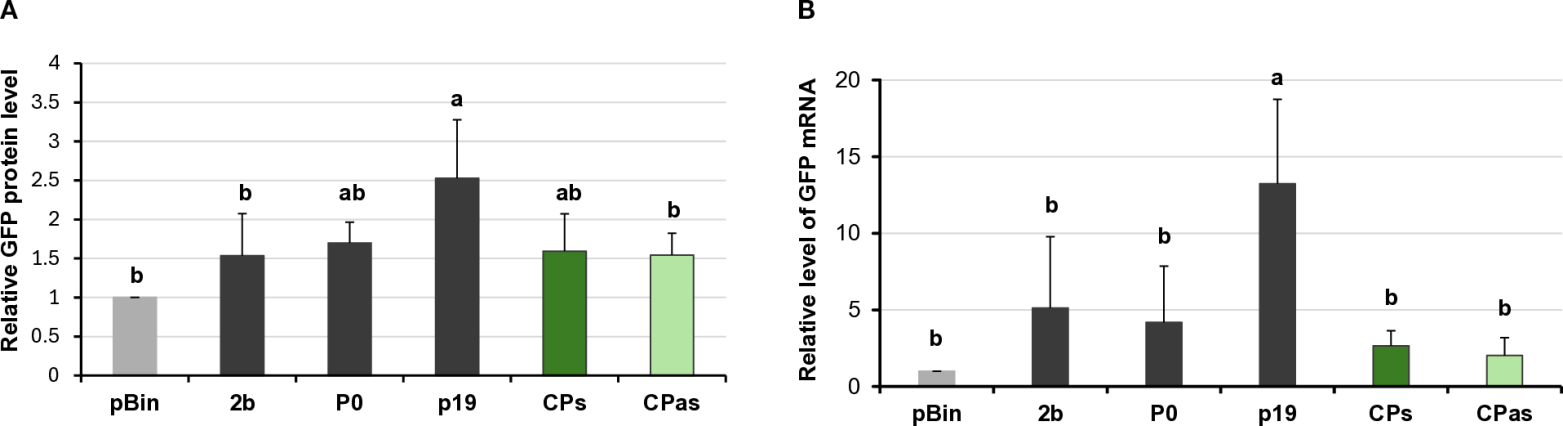
Quantifying the GFP protein and GFP mRNA levels in the transient silencing assay when the VSR activity of the GPGV-CP was tested. **(A)** Column diagram of the GFP protein level quantified using Western blot experiments. The sample signal was normalised to the pBin empty vector/GFP signal (as negative control). p19^CymRSV^, 2b^CMV^, and P0^BWYV^ were used as positive controls. Mean values were calculated from three independent experiments. The error bars indicate the standard error, *n* = 3. Letters indicated significant difference at the 0.05 level according to one-way ANOVA and Tukey HSD test. **(B)** GFP mRNA level quantified using quantitative RT-PCR using the 2^−△△Ct^ method and the expression of the *N. benthamiana* ubiquitin gene as the internal control. The sample signal was normalised to the pBin empty vector/GFP signal (as negative control). p19^CymRSV^, 2b^CMV^, and P0^BWYV^ were used as positive controls. The error bars indicate a standard error, *n* = 3 experiments. Letters indicated significant difference at the 0.05 level according to one-way ANOVA and Tukey HSD test.

Investigating the potential VSR activity of the proteins encoded by a symptomatic variant of GPGV (fvg-ls12 – MH087443), the CP showed local silencing suppressor activity (Tarquini et al., 2021b), but the possible VSR activity of the asymptomatic variant (fvg-ls15 – MH087446), identified by the same group (Tarquini et al., 2019a, 2019b), was not tested. Similar to Tarquini and colleagues, we found symptomatic (MK953677_HUCSK9s) and asymptomatic (MK953676_HUCSK8as) variants of GPGV at a Pinot gris vineyard at Csókakő in Hungary (Demian et al., 2020). We wondered whether the HUCSK9s strain has similar local VSR activity as the fvg-ls12 virulent strain. Moreover, we were curious if there is any detectable difference between the local VSR activity of CPs encoded by the symptomatic and asymptomatic GPGV variants. The local VSR activity of the GPGV-CPs (CP of the HUCSK9s) and GPGV-CPas (CP of the HUCSK8as) was compared using a standard GFP-based test (Voinnet and Baulcombe, 1997; Voinnet et al., 1998). In this transient assay, leaves of wt *N. benthamiana* plants were infiltrated with a mixture of *Agrobacterium* cultures expressing GFP and the GPGV-CP proteins. As a positive control, *Agrobacterium* expressing previously characterised VSR proteins: p19^CymRSV^, 2b^CMV^, and P0^BWYV^, while as a negative control empty binary vector transformed *Agrobacterium* was used. Protein coding capacity of the HA-tagged constructs showed that the tested proteins were expressed in a comparable quantity (**Supplementary Figure S6A**). In the transient assay, GFP fluorescence of the GPGV-CP infiltrated leaves was compared to the fluorescence intensity of the control at 4 dpi (**Figure 2**). In the presence of the GPGV-CP of both the symptomatic and asymptomatic variants, the GFP fluorescence was slightly stronger than in the presence of the empty vector (control), indicating their weak, local VSR activity (**Figure 2**), supporting the original result of Tarquini (Tarquini et al., 2021b). Quantification of the GFP protein levels determined by Western blot (**Supplementary Figure S7**; **Figure 3A**) and GFP mRNA levels, determined by RT-qPCR (**Figure 3B**), confirmed this observation. This local VSR activity of the GPGV-CP was slightly stronger, but not statistically different (p-value: 0,99), in the case of the symptomatic variant.

**Figure 4:**
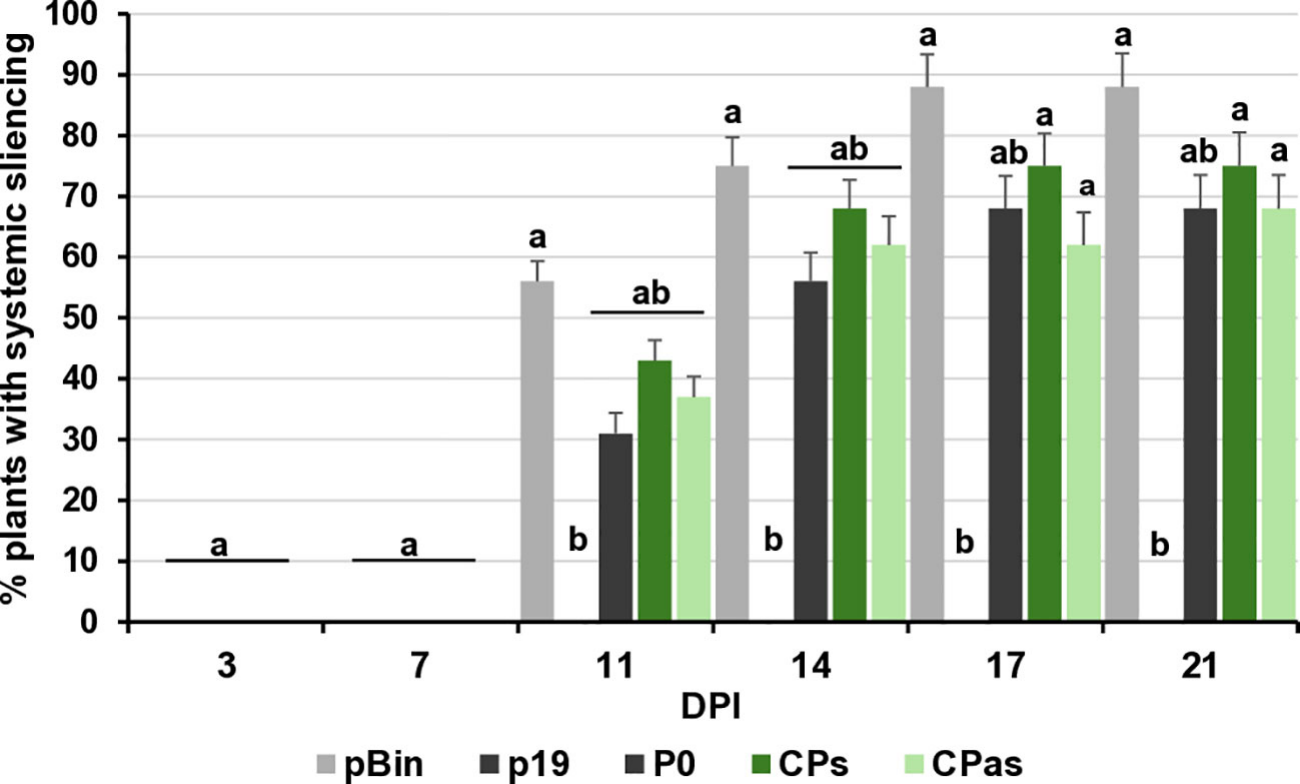
Summarised result of the transient silencing assay-based test of the systemic VSR activity of the GPGV-CP encoded by the symptomatic and asymptomatic GPGV variants. Time dependence of the systemic silencing scores during the experiment. The error bars indicate a standard error. Letters indicated significant difference at the 0.05 level according to one-way ANOVA and Tukey HSD test.

VSRs can not only suppress the RNAi processes locally, but they can also interfere with the movement of the silencing signal and have systemic silencing activity. This type of activity of GPGV-CP has not been tested before. To evaluate whether GPGV-CPs and/or GPGV-CPas can interfere with systemic silencing, leaves of GFP-expressing transgenic 16c *N. benthamiana* were infiltrated with a mixture of GFP and p19^CymRSV^ (strong systemic silencing inducer), P0^BWYV^ (having no systemic silencing activity), and the GPGV-CP, encoded by the symptomatic (ORF3s) or the asymptomatic (ORF3as) variant. In the 16c GFP-expressing plant, the colour of the leaves and stems is a pale red, because the chlorophyll autofluorescence is masked by the green colour of the expressed GFP protein. When, because of RNAi, the GFP expression is blocked, the deep red colour becomes visible. At 7 dpi, similarly to the negative controls (pBin and P0), we observed the formation of the “red halo” in the infiltrated leaves, indicating the lack of systemic VSR activity of the GPGV-CP (**Supplementary Figure S8**). Monitoring the spread of the silencing signal to long distances, detected as red veins, red patches of the leaves, GFP expression in the non-infiltrated, systemic leaves was observed for 21 days, and the number of plants showing systemic silencing was counted (**Supplementary Figure S9**). We observed that the systemic silencing appeared quickly and in the majority of the tested plants. Similarly to the negative controls (pBin - 88% and P0 - 68%) the percentage of the silenced plants was 75% and 68% in the case of GPGV-CPs and GPGV-CPas, respectively. What was in striking contrast to the positive control (p19 – 0%), where the systemic silencing was delayed or did not appear at all, indicating that GPGV-CP has no systemic silencing activity (**Figure 4**; **Supplementary Table 3**).

Unrelated VSR could translationally inhibit AGO1 activity through their miR168 induction (Várallyay et al., 2010; Várallyay and Havelda, 2013). To test whether this mechanism also exists in the case of the GPGV-CP, the miR168 level in the infiltrated patch was quantified using a small RNA Northern blot assay. We found that expression of GPGV-CPs and GVPV-CPas did not alter the miR168 expression levels, suggesting that AGO1 regulation through miR168 induction is not the mechanism in the case of GPGV-CP VSR activity (**Supplementary Figure S10**). Taken together, our results demonstrated that GPGV-CP has a local but not systemic silencing activity.

### 3.3 GPGV-ORF2 has slight systemic silencing activity

Tarquini and colleagues investigated the possible VSR activity of all three GPGV-ORF-encoded proteins (Tarquini et al., 2021b). They only identified local VSR activity in the case of GPGV-ORF3 (CP) encoded by the virulent variant. To be able to conclude that neither local nor systemic silencing activity of the MP encoded by ORF2 of the virus exists, we tested the local and systemic silencing activity of GPGV-MPs (MP of the HUCSK9s) and GPGV-MPas (MP of the HUCSK8as) using the transient VSR assay.

**Figure 5:**
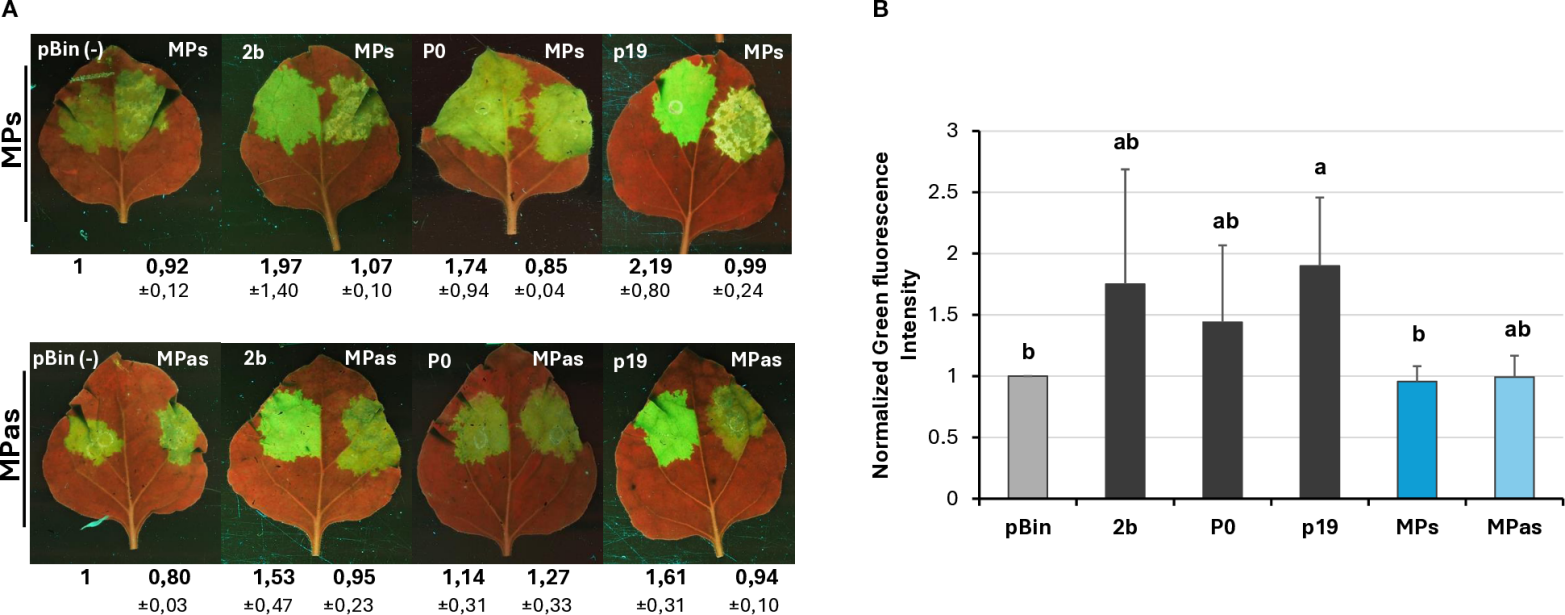
Transient silencing assay-based test of the local VSR activity of the GPGV-MP encoded by the symptomatic and asymptomatic GPGV variants. **(A)** Representative photos of leaves of wt *N. benthamiana* coinfiltrated with GPF and GPGV-MP (upper panel – MPs, lower panel – MPas). The empty vector (pBin) was used as a negative; p19^CymRSV^, 2b^CMV^, and P0^BWYV^ were used as positive controls. Photos were taken at 4 dpi under UV light. Each experiment was repeated three times. The fluorescence intensity of the infiltrated zones was quantified using ImageJ software. Bold numbers show the average fluorescence intensity of all of the infiltrated leaves of all three experiments, and ± indicates standard deviation. **(B)** Fluorescence intensity of the three independent experiments. The error bars indicate the standard deviation. Letters indicated significant difference at the 0.05 level according to one-way ANOVA and Tukey HSD test.

**Figure 6:**
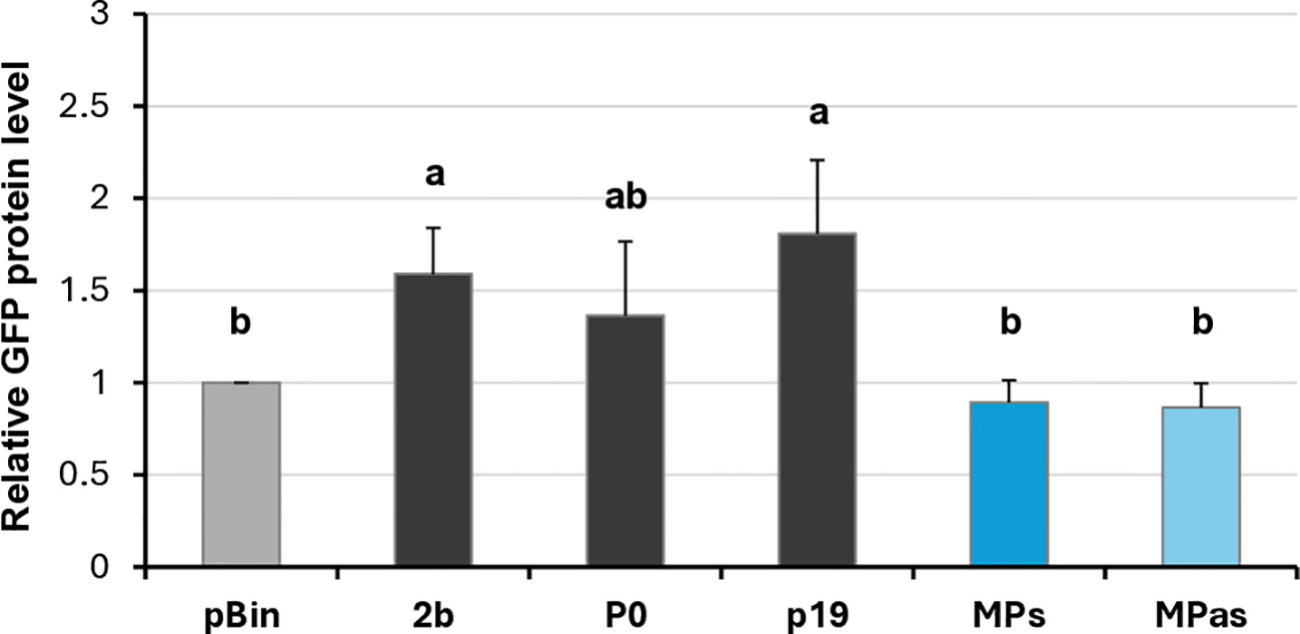
Quantifying the GFP protein levels in the transient silencing assay when the VSR activity of the GPGV-MP was tested. GFP protein level quantified using Western blot experiments. The sample signal was normalised to the pBin empty vector/GFP signal (as negative control). p19^CymRSV^, 2b^CMV^, and P0^BWYV^ were used as positive controls. Mean values were calculated from three independent experiments. The error bars indicate a standard error, *n* = 3. Letters indicated significant difference at the 0.05 level according to one-way ANOVA and Tukey HSD test.

Changes in the GFP fluorescence activity and the GFP protein level in the GPGV-MP infiltrated zones confirmed that although the protein is expressed correctly (**Supplementary Figure S6B**), the GPGV-MP had no local silencing activity (**Figure 5**; **Supplementary Figure S11**; **Figure 6**). To test the possible systemic silencing activity of the GPGV-MP, the infiltration assay was conducted in GFP-expressing *N. benthamiana* 16c plants, and the appearance of the silencing was monitored. At 7 dpi, in the GPGV-MP-infiltrated leaves, we detected a “red halo”, which normally reports the initial cell-to-cell movement of the signal from the infiltrated patch (**Supplementary Figure S8**), indicating that at a short distance, the silencing signal was not blocked by either form of the GPGV-MP.

**Figure 7:**
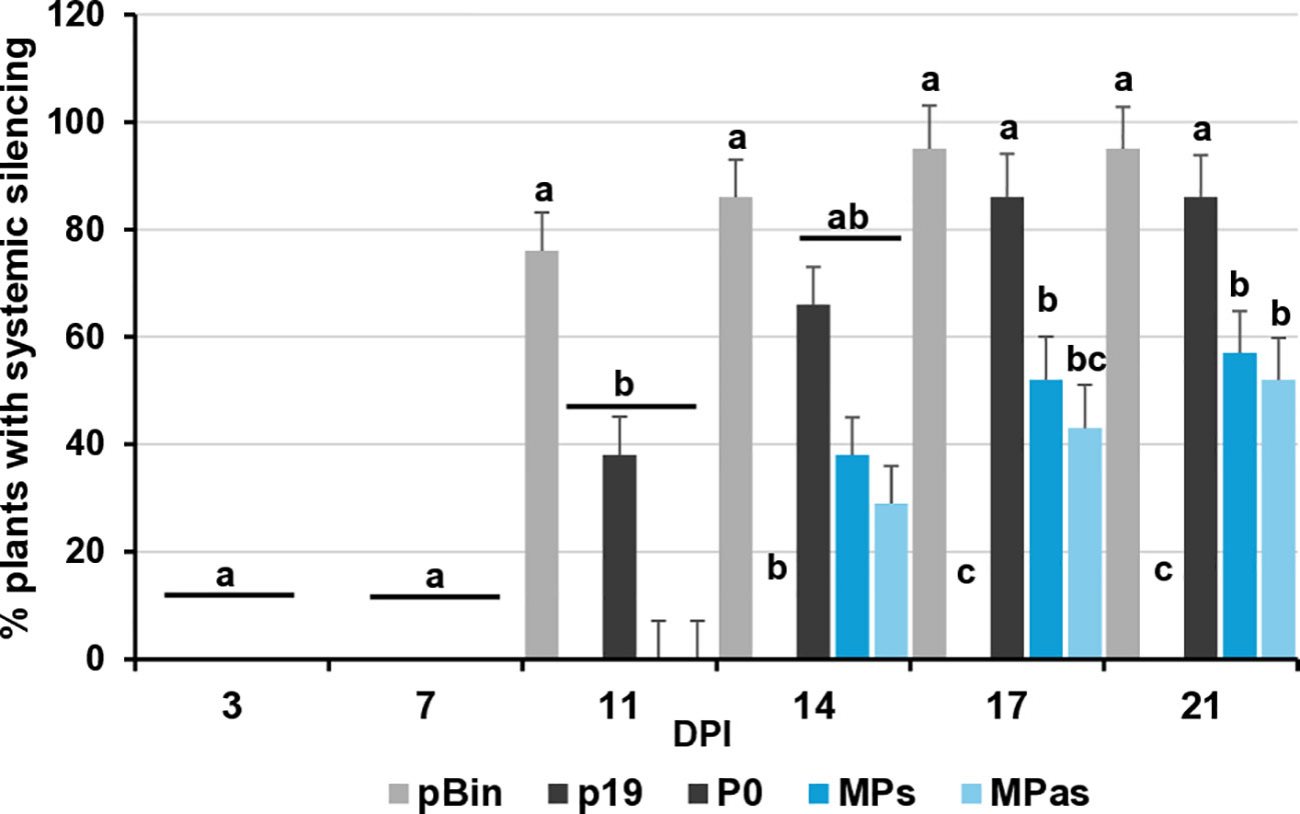
Summarised result of the transient silencing assay-based test of the systemic VSR activity of the GPGV-MP encoded by the symptomatic and asymptomatic GPGV variants. Time dependence of the systemic silencing scores during the experiment. The error bars indicate a standard error. Letters indicated significant difference at the 0.05 level according to one-way ANOVA and Tukey HSD test.

At 21 dpi, GFP fluorescence was observed in the upper leaves under UV light (**Supplementary Figure S12**). Appearance of the silencing was detected in 66% and 52% of the plants infiltrated with GPGV-MPs and GPGV-MPas, respectively, which were statistically less than in the case of the negative control. Moreover, monitoring the leaves of these plants for silencing signal showed that not only was the number of plants showing systemic silencing statistically lower than in the case of the negative control, but also the number of silenced leaves followed this trend (**Figure 7**; **Supplementary Table 4**). Our results indicate that even though GPGV-MP has no local VSR activity, it has a moderate systemic silencing VSR activity. This GPGV-MP VSR activity is not statistically different between the symptomatic and asymptomatic variants.

### 3.4 GPGV-MP variants caused necrosis on *N. benthamiana* leaves

**Figure 8:**
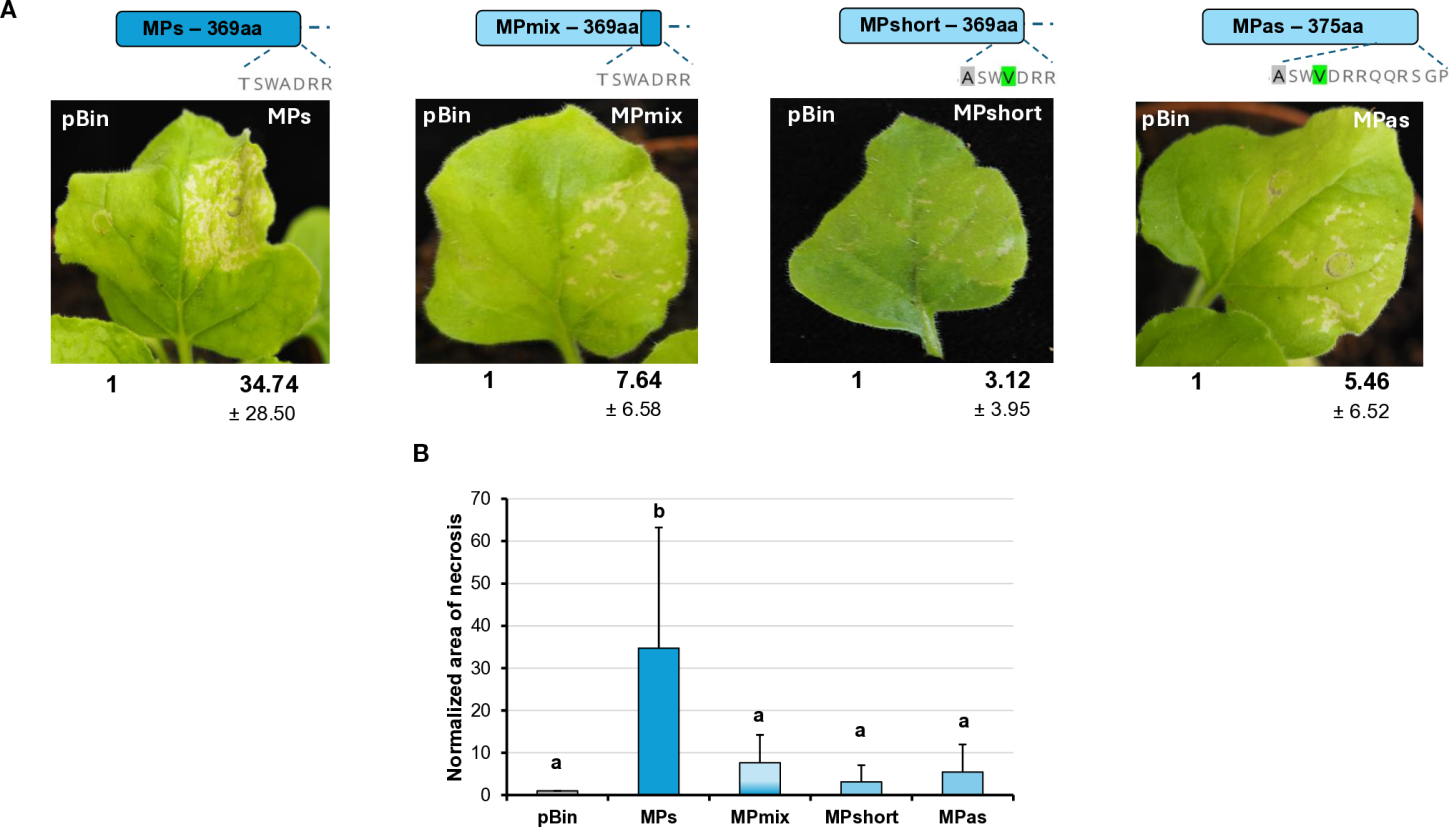
Transient expression of the GPGV-MP induces necrosis I. **(A)** Representative photographs of wt *N. benthamiana* leaves infiltrated with different GPGV-MP variants at 5 dpi after agroinfiltration. The empty vector pBin was used as a negative control. Each experiment was repeated three times. Bold numbers show the quantification of necrotic area measurement (mean necrotic area) of all experiments using ImageJ software (± indicates standard deviation, SD). The schematic diagram of the constructs is shown, indicating the differences in the backbone. Light blue represents the asymptomatic, while dark blue, the symptomatic variant. The last 7–13 amino acids at the 3’ end of the protein are also shown, highlighting the differences between the constructs. **(B)** The mean necrotic areas of the infiltrated leaves. Each experiment was repeated three times, infiltrating two leaves of 10 plants each time. The error bars indicate the standard deviation. Letters indicated significant difference at the 0.05 level according to one-way ANOVA and Tukey HSD test.

During the *Agrobacterium* infiltration assay, we observed that transient GPGV-MP expression induced necrosis in the infiltrated tissues from 4 dpi. In the case of GPGV-MPas, the necrosis was detected in fewer leaves with milder severity than in the case of GPGV-MPs (**Figure 8A**).

The most different part of the symptomatic and asymptomatic GPGV-MP protein is the vicinity of the 370 amino acid position at the 3’ end of the protein. In this region at the carboxy-terminal end of the MP, HUCSK8as and HUCSK9s differ only at two additional positions: Ala363 is Thr, while Val366 is Ala in the symptomatic variant (**Supplementary Figure S5**). To test whether these changes located in the carboxy-terminal of the GPGV-MP could play a role in the necrosis induction, recombinant MPs have been generated. GPGV-MPshort is an MP of the asymptomatic strain, which was edited to be six-amino-acid shorter. GPGV-MPmix is an edited version of the GPGV-MPshort, containing Ala363Thr and Val366Ala mutations, similar to the GPGV-MPs. Results from three independent infiltration experiments testing two leaves from 10 plants showed that the necrosis of these two mutants was less severe and more similar to the necrosis caused by GPGV-MPas (**Figure 8B**).

To further localise which region of the MP is responsible for necrosis induction, we tested the necrosis-inducing activity of MPs encoded by an irregular natural GPGV variant. The HU-27 variant (originating from Hungary), despite its long MP, clusters with the symptomatic clade (Sáray et al., 2024) (**Supplementary Figure S3**). Previously, only the MP/CP coding polymorph region of this clone was sequenced. In order to test its necrosis-inducing activity, we determined the full MP sequence (GenBank accession number: PV208197).

**Figure 9:**
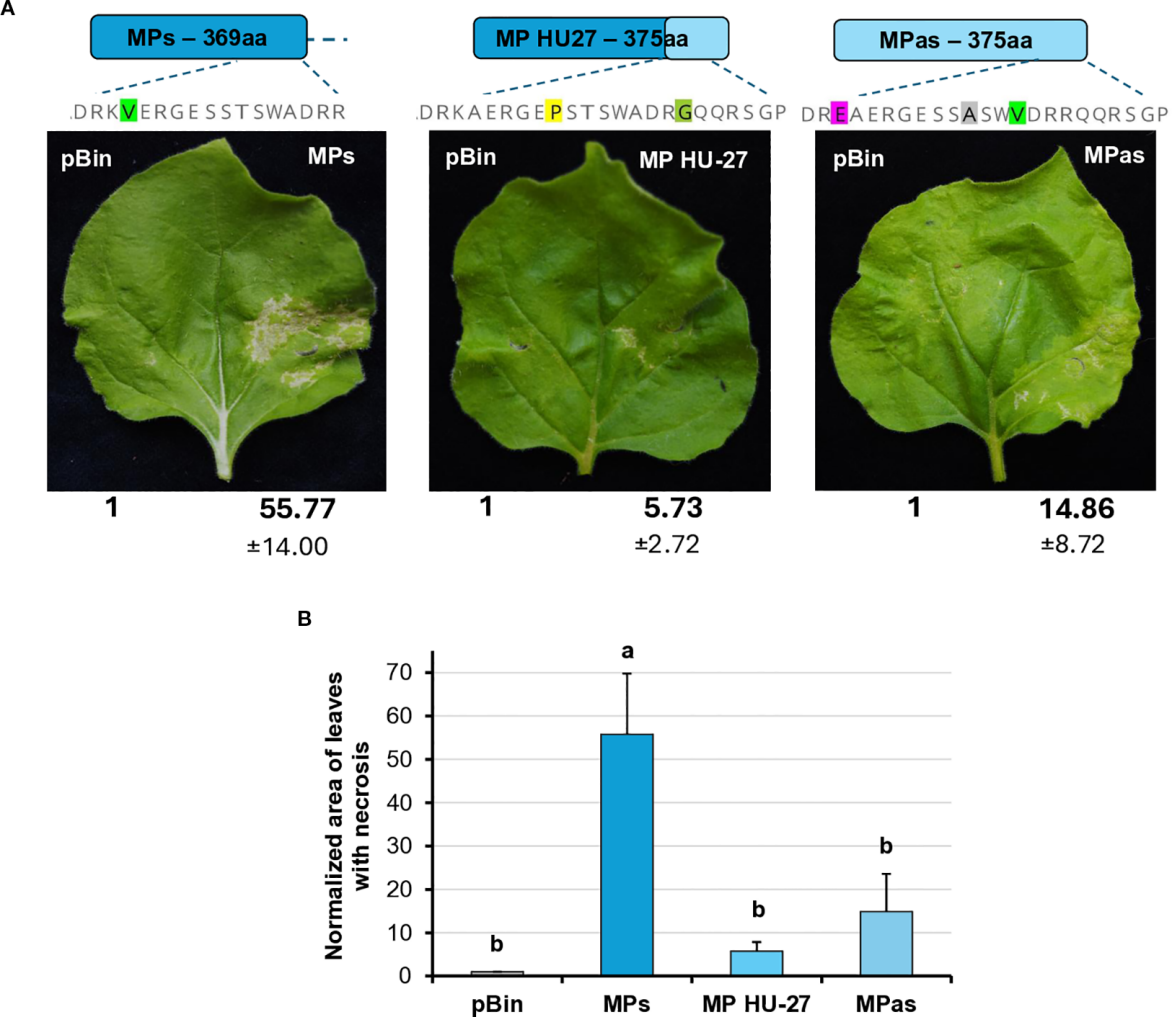
Transient expression of the GPGV-MP induces necrosis II. **(A)** Representative photographs of wt *N. benthamiana* leaves infiltrated with different GPGV-MP variants at 5 dpi after agroinfiltration. The empty vector pBin was used as a negative control. Each experiment was repeated three times. Bold numbers show the quantification of necrotic area measurement (mean necrotic area) of all experiments using ImageJ software (± indicates standard deviation, SD). The schematic diagram of the constructs is shown, indicating the differences in the backbone. Light blue represents the asymptomatic, while dark blue, the symptomatic variant. The last 17–23 amino acids at the 3’ end of the protein are also shown, highlighting the differences between the constructs. **(B)** Mean necrotic areas of the infiltrated leaves. Each experiment was repeated three times, infiltrating two leaves of 10 plants each time. The error bars indicate the standard deviation. Letters indicated significant difference at the 0.05 level according to one-way ANOVA and Tukey HSD test.

The transient assay of GPGV-MP HU-27 revealed that the necrotic symptoms were milder than in the case of either the MPs or MPas (**Figure 9**). Although the number of plants in which the MP HU-27 variant induced necrosis was approximately the same as in the case of GPGV-MPs, the severity of the necrotic symptoms was much weaker.

We intended to test whether this necrotic-inducing activity of the GPGV-MP is present in other hosts, but our several attempts for successful transient protein expression using the *Agrobacterium*-based transient assays in grapevine failed. However, transient assays performed in *N. tabacum* cv. Xanthi and *N. glutinosa* revealed that no necrosis was induced in *N. tabacum* cv. Xanthi (**Supplementary Figure S13**), but did in *N. glutinosa* (necrotic symptoms in this host were less pronounced than in the highly susceptible *N. benthamiana*) (**Supplementary Figure S14**).

These results confirmed that GPGV-MP is capable of inducing necrosis in a different host, and that this necrosis-inducing activity was most pronounced in GPGV-MPs, with much milder necrosis observed in the other GPGV-MP variants (GPGV-MPas, GPGV-MPShort, GPGV-MPmix and GPGV-MP HU-27).

### 3.5 Investigation of the structure of GPGV-MP to locate key elements for the difference between the symptom-inducing activity of symptomatic and asymptomatic variants

**Figure 10:**
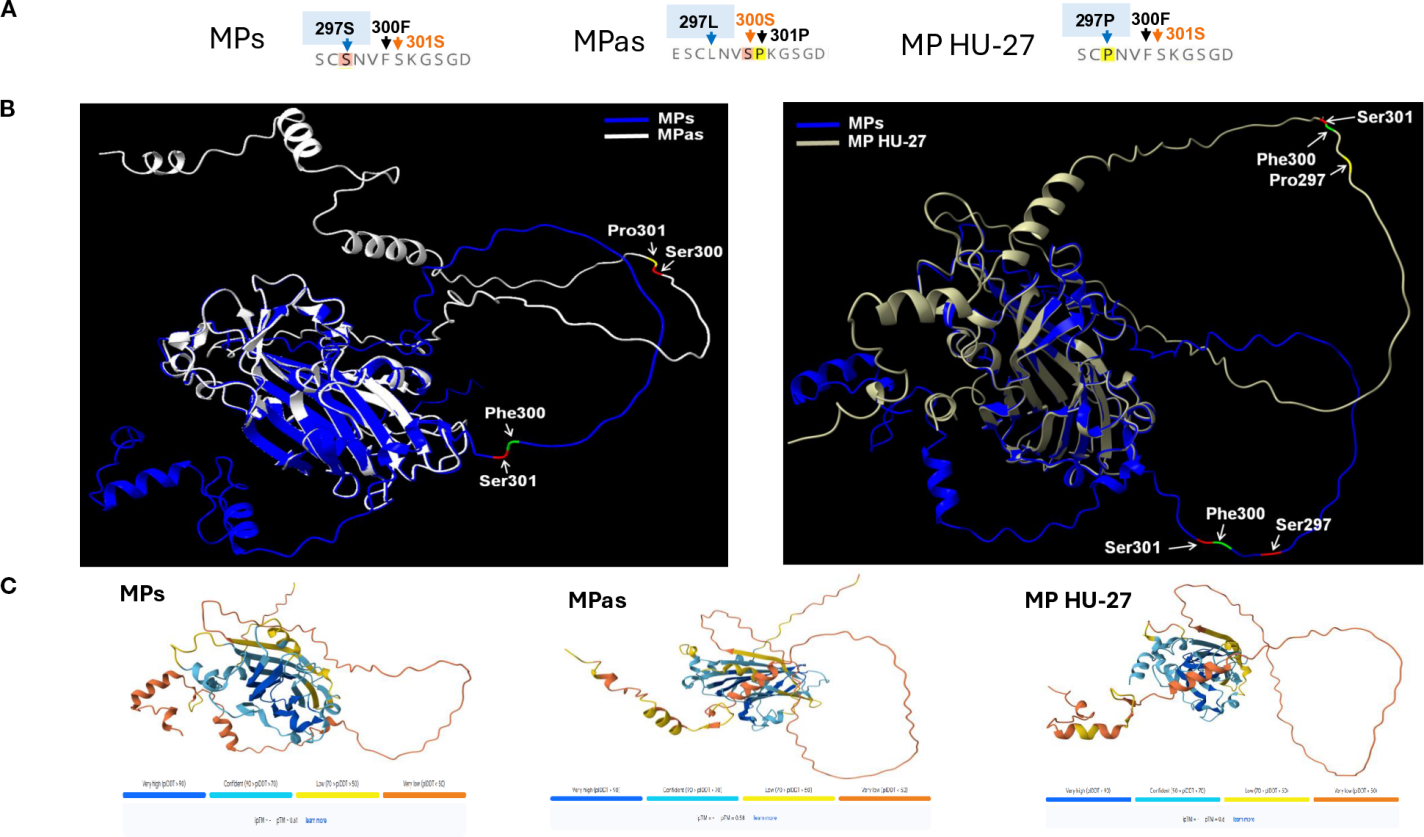
The predicted structure of GPGV-MP variants. **(A)** Amino acid sequence of the three main MP protein variants at the 294–306 region. **(B)** The predicted MP protein structures and locations of amino acid differences comparing the MPs and MPas (right panel) and MPs and MP HU-27 variants (left panel). ChimeraX-Alphafold3 protein structure prediction was used to generate the figures. **(C)** Alphafold3 is used for the prediction of MP protein structure. The image below indicates the confidence scores in the projected conformations. The confidence level of AlphaFold3’s predictions varies for each protein. Dark blue and light blue regions on a predicted structure mean the algorithm is relatively confident. Less certain predictions are coloured yellow and orange.

To analyse the complete MP region encoded by the five GPGV-MP variants used, we carried out multiple protein sequence alignments and structure prediction analysis. The result of this analysis allowed us to locate the presence of several amino acid alterations, mostly at the 3’ end of MP (**Supplementary Figure S5**). We found that the backbone structure of the variants (GPGV-MPs, -MPas, and -MP HU-27) are the same. 3D structure prediction is based on homology modelling, physicochemical principles, or energy minimization approaches. However, disordered structures usually do not have homology, and the reliability of their sequence prediction based solely on calculations is less reliable. The reliability of the predicted structure for the GPGV-MP carboxy-terminal part (including variations at positions 297, 300, and 301) is much lower than that of the rest of the protein; however, variations at these positions, especially changes in polarity or hydrophobicity, could still affect the protein structure (**Figure 10**). In the GPGV-MPs and GPGV-MPas variants, there are two closely adjacent amino acid changes at positions 300 (S_as_/F_s_) and 301 (P_as_/S_s_) on a not structured surface loop, which could function as an interaction site. At 297, the MPs variant contains serine, while MPas and MP HU-27 code for proline, which may alter the protein structure. The polar serine at position 300 in MPas is replaced by a phenylalanine, a residue with strong hydrophobic character, potentially affecting the loop structure. This effect may be further altered by the neighbouring amino acid at position 301. Here, serine is present in the GPGV-MPs and HU-27, which is replaced by a rigid proline in the case of GPGV-MPas, which can induce twists and disruptions of the proper structure, altering the protein-protein interactions and consequently the function of the protein (**Figure 10**).

Besides protein structure changes at potential interaction sites, post-translational modifications can alter the function of proteins. The possible phosphorylation sites of the investigated GPGV-MP variants were predicted. Out of the several predicted phosphorylation sites, only one showed a characteristic difference between the asymptomatic and symptomatic variants (**Supplementary Table 5**; **Supplementary Figure S15**). The serine located at position 300 of the GPGV-MPas was predicted to be phosphorylated, while this possible phosphorylation site is missing in the GPGV-MPs and MP HU-27 variants, where phenylalanine is present at this position (**Supplementary Figures S5**, **S15**). Alteration of the MP structure and its possible phosphorylation could affect its proper interaction with the host proteins, which could be different in different hosts. GPGV was detected in some non-*Vitis* hosts: *Ailanthus, Asclepias, Crataegus, Fraxinus, Rosa, Rubus, and Sambucus* species (Demian et al., 2022). The program predicted a serine-phosphorylated site at position 300 aa only in *Ailanthus* (ON360690), while in the other non-*Vitis*-GPGV variants, phenylalanine is present at this position, and this potential phosphorylation site is missing (**Supplementary Figures S5**, **S15**).

## 4 Discussion

During evolution, viruses have evolved to encode one or more silencing suppressor proteins to defeat the well-established RNAi-based antiviral defence of the host plant (Csorba et al., 2015). The presence and strategy of the VSR can define how the virus suppresses the host defence mechanisms, which are key factors determining the development of virus-specific symptoms.

The involvement of GPGV in the development of GLMD is still not clear. Tarquini and co-workers (Tarquini et al., 2021b) have shown that the CP of the virus (GPGV-ORF3) suppresses host antiviral silencing, but they tested only the CP encoded by the symptomatic fvg-ls12 isolate. The asymptomatic variant sequenced in that study is fvg-ls15, whose 3’ end sequence is missing (**Supplementary Figure S4**). Although variations at the fvg-ls15 carboxy-terminal (missing) amino acids are possible, the sequenced part of the CP encoded by this asymptomatic variant and fvg-ls12 is identical, explaining why VSR activity of only one variant was tested. In our work, we confirmed and complemented these results. We showed that GPGV-CP encoded by another symptomatic (HUCSK9s) and an asymptomatic (HUCSK8as) variant were weak VSRs, blocking local but not systemic RNAi. The CPs encoded by these variants differ both from the fvg-ls12 variant encoded CP and from each other at positions 64 and 96 (**Supplementary Figure S4**). These amino acid substitutions could slightly affect the structure of the CP and its interaction with the interacting host factors. Additionally, other effects related to the nucleotide sequence, such as the interaction with transcriptional or post-transcriptional factors, should not be ruled out.

Although the VSR activity of the GPGV-CP has been identified, the molecular mechanism of the silencing process has not been investigated in detail. In GPGV-infected *N. benthamiana* plants, Tarquini et al. (2021b) hypothesised that the possible way of GPGV-CP VSR action is the inhibition of AGO1. Although we did not verify the AGO1 level, the change in the miR168, which is responsible for the translational inhibition of AGO1, was found to be constant in the infiltrated zones using a small RNA Northern blot assay. In their work, Tarquini and colleagues quantified the gene expression changes of the key enzymes of the RNAi pathway after infecting *N. benthamiana* with the virulent virus. As no results about the changes in the expression pattern of these key elements of RNAi are available in the case of the latent virus infection, we think that the weak VSR activity of GPGV-CP is important during GPGV infection, but no definitive conclusions can be drawn regarding its involvement in symptom development.

Besides slight local VSR activity of the CP, we detected a slight systemic silencing activity of the GPGV-ORF2 (MP) encoded protein. Although it has not been proven, based on similarity to other trichoviruses, the GPGV-ORF2 encodes the viral MP. The MP’s function in the viral infection cycle is assisting in the cell-to-cell movement of the virus (Amari et al., 2012; Han et al., 2019; Navarro et al., 2019), but it can have additional functions. Encoded in the ortholog position to the GPGV-MP, the P50 protein of another trichovirus, apple chlorotic leaf spot virus (ACLSV), was identified as a VSR and lacked local but showed systemic silencing activity (Yaegashi et al., 2007; Kumar and Dasgupta, 2021). The local VSR activity of the GPGV-CP and the systemic VSR activity of the GPGV-MP differed slightly, but not statistically, which is why it might not be involved in the difference in symptom development during infection with these different strains. In this study, we observed that although GPGV-MP did not inhibit the silencing processes locally, its transient expression induced local necrosis. Necrosis-inducing activity of the VSRs has been observed in several cases: 126K replication protein of tobamoviruses and P0 could also induce necrosis on the infiltrated leaves (Ding et al., 2004; Mangwende et al., 2009; Fusaro et al., 2012). In the transient test, we observed local necrosis on the test plant, indicating that the presence of the protein interfered with and disrupted the cellular functions. We found that the necrosis-inducing activity of the symptomatic strain was more severe and hypothesised that this behaviour of a viral protein can affect the symptom development during virus infection. Modifications in the 3’ region of MP demonstrated differences in their necrosis-inducing activity. Altering the length of the MP and the presence of some differentiating amino acids at the 3’ end of the CP resulted in decreased necrosis-inducing activity, regardless if the backbone of the protein was derived from the symptomatic (MP HU-27) or the asymptomatic variants (MPshort), indicating that the presence of certain amino acids at the 3’ end of the MP could play important role in this activity. However, we have found that MPmix, a short MP version having amino acids specific to the symptomatic clade, has lost this severe necrosis-inducing activity, which suggests that certain amino acid positions, besides the C-terminus of the protein, can also play an important role in the ability to induce necrosis.

Variation of amino acids at certain points of the proteins may affect protein function and may be responsible for the development of symptoms. Comparison of the amino acid sequence of the GPGV-MP revealed several differentiating positions which are characteristic of the symptomatic or the asymptomatic variants (**Figure 1**; **Supplementary Figure S5**). Among them, amino acids around position 300 can be important, as here, changes from leucine or serine to proline can be found. Introduction of proline into the protein backbone usually causes a break in its well-defined secondary structure and disrupts or alters the protein’s interaction with its interacting partners. We found that the backbone structure of the GPGV-MP variants is relatively stable based on confidence score, but we got uncertain predictions in the case of the carboxy-terminal part of the MP. Position 300 aa of the different strains can be found at different parts in a loop, with an altered angle to the rest of the protein, which could lead to an altered interaction potential with other proteins (**Figure 10**). Around this position, we have located important differences: positions 297, 300, and 301. Here altered presence of Ser and Pro can affect the possible phosphorylation and may affect the loop structure (**Figure 10**).

Phosphorylation of MP has been shown to occur in several viruses, such as tobacco mosaic virus (Haley et al., 1995), tomato mosaic virus (Kawakami et al., 1999), potato leafroll virus (Sokolova et al., 1997), and cucumber mosaic virus (Matsushita et al., 2002; Sáray et al., 2021). In our work, an *in silico* study of the phosphorylation sites of GPGV-MP variants showed that there is a potential difference in the phosphorylation of the variants. At position 300 aa, serine or phenylalanine can be present, but only serine (present in the asymptomatic variants) can be phosphorylated.

In conclusion, we confirmed the local VSR activity of the GPGV-CP, but did not find statistical differences between the symptomatic and asymptomatic variants. In addition, GPGV-ORF2 encoded MP showed a moderate systemic VSR activity and induced necrosis on the infiltrated leaves, which was more severe in the case of the symptomatic variant. The association of the symptom developmental potential with the C-terminal sequence of GPGV-MP has been demonstrated experimentally (Tarquini et al., 2021a; Karki et al., 2025), but the link between genomic variations and symptom manifestation requires further investigation. We suggest that, in addition to the presence of an early stop codon, the presence of certain amino acids and phosphorylation around position 300 aa can also contribute to the development of GLMD symptoms. Although this hypothesis can be tested not only in transient assays, but also using infectious clones of recombinant viruses, on the grapevine, we cannot rule out that it will only partially alter the symptom development, which could also be affected by several biotic and abiotic stress factors.

## Data Availability

The datasets presented in this study can be found in online repositories. The names of the repository/repositories and accession number(s) can be found below: https://www.ncbi.nlm.nih.gov/genbank/, PV208197.
