# Supplementary material for: Comparative functional analyses of the movement and coat proteins of grapevine Pinot gris virus, encoded by symptomatic and asymptomatic variants
Source: Front Plant Sci. 2025 Sep 25;16:1659802. doi: 10.3389/fpls.2025.1659802 (PMC12507868; doi:10.3389/fpls.2025.1659802)
Supplement: Supplementary file 1 [file DataSheet1.pdf]

Supplementary Figures

Comparative functional analyses of the movement and coat proteins of grapevine Pinot gris virus, encoded by symptomatic and asymptomatic variants

Nikoletta Jaksa-Czotter[1]*, Emese Demián[2], Réka Sáray[2], Katalin Salánki[2], Éva Várallyay[1]*

[1]Genomics Research Group, Department of Plant Pathology, Institute of Plant Protection, Hungarian University of Agriculture and Life Sciences, Hungary
[2]Department of Plant Pathology, Plant Protection Institute, Centre for Agricultural Research, HUN-REN, Hungary

*Corresponding authors: jaksa-czotter.nikoletta@uni-mate.hu and varallyay.eva@uni-mate.hu

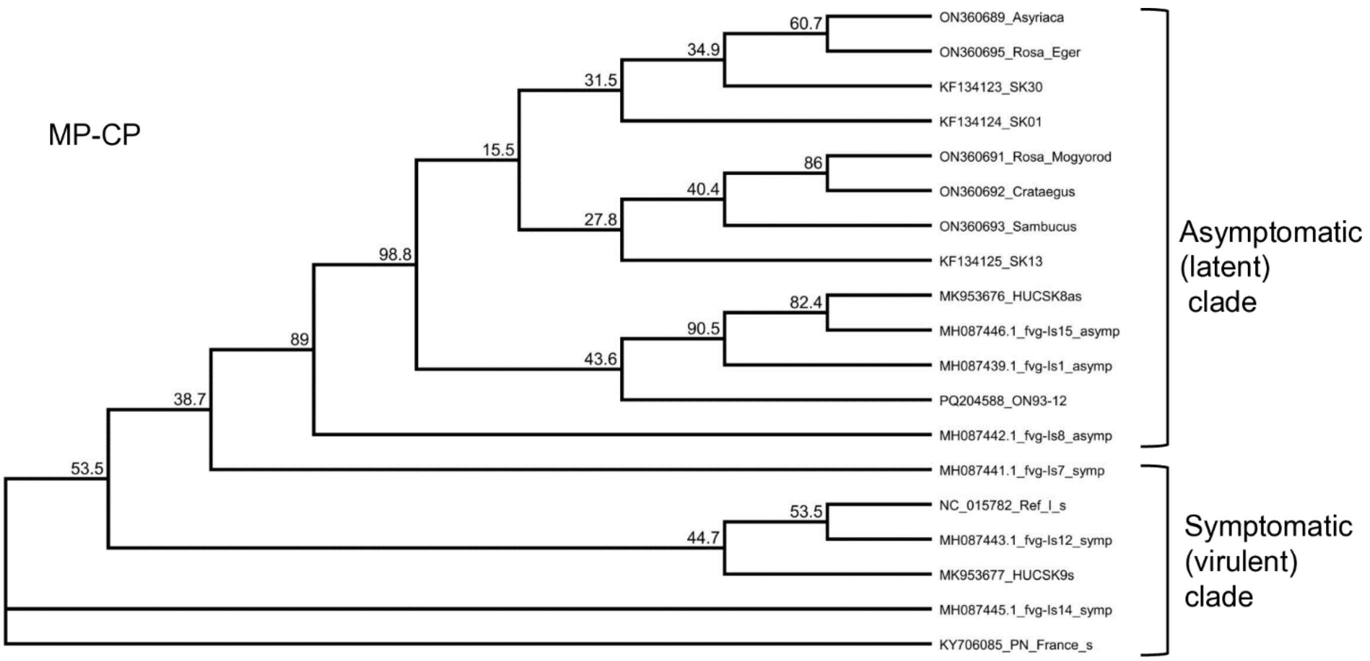

Figure S1.
Phylogenetic analysis based on the full MP and CP coding regions of the GPGV variants discussed in this manuscript. The alignment was prepared using the MUSCLE algorithm in Geneious Prime (version 2024.0.7). The tree was constructed using Geneious Tree Builder, using the Jukes-Cantor model and the Neighbour-Joining method, using 1000 bootstrap replicates.

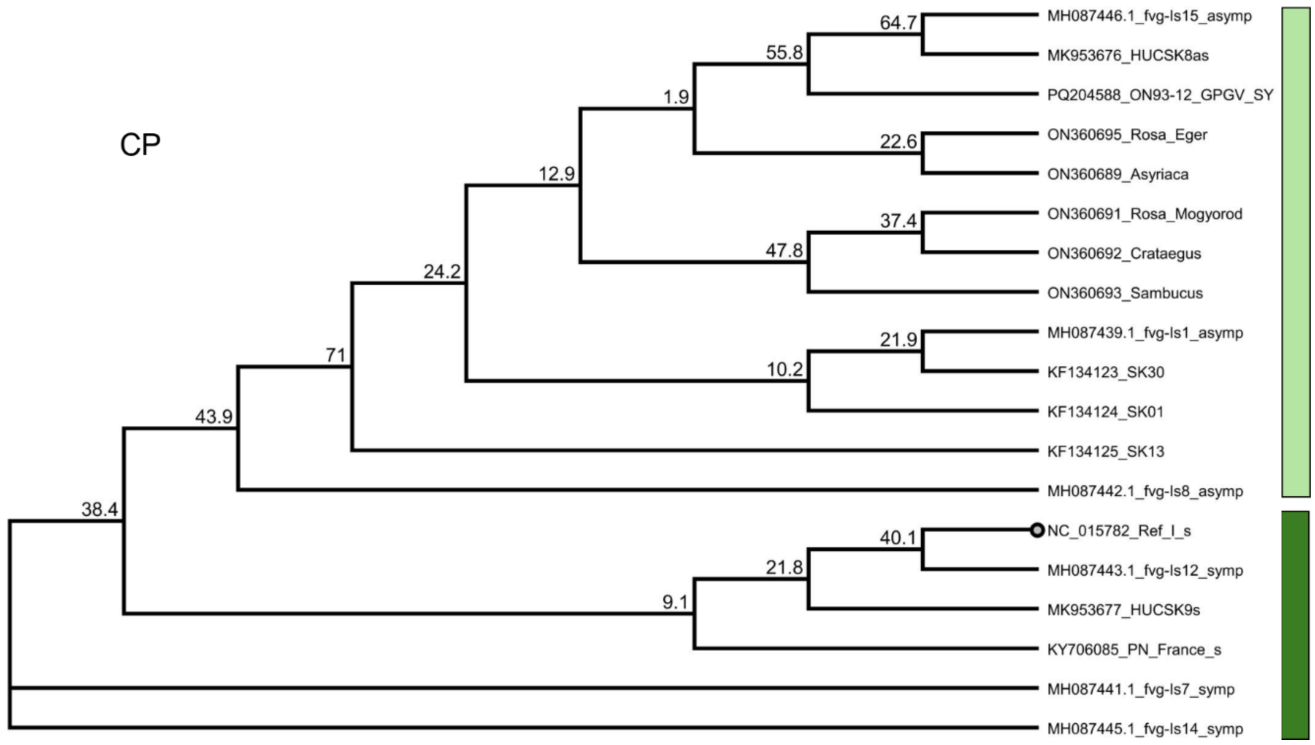

Figure S2.

Phylogenetic analysis based on the CP coding regions of the GPGV variants discussed in this manuscript. The alignment was prepared using the MUSCLE algorithm in Geneious Prime (version 2024.0.7). The tree was constructed using Geneious Tree Builder, using the Jukes-Cantor model and the Neighbour-Joining method, using 1000 bootstrap replicates. Light green indicates the asymptomatic, while dark green indicates the symptomatic clade.

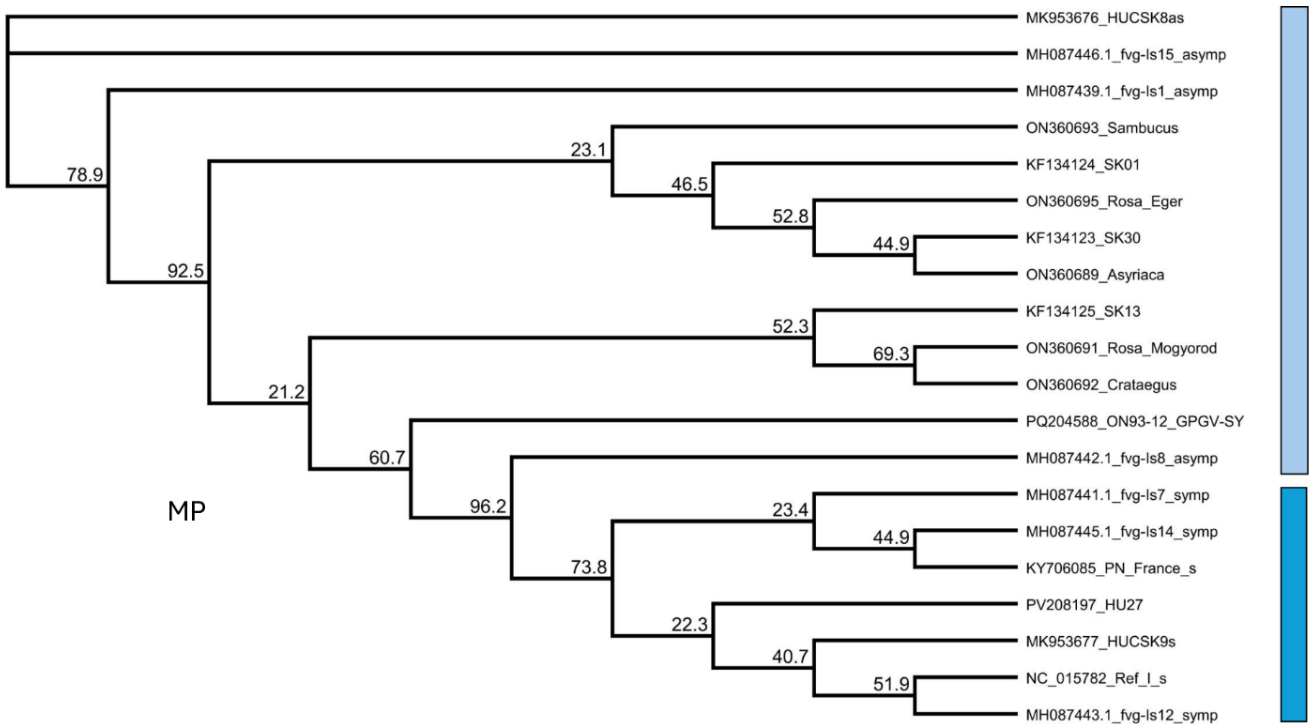

Figure S3.
Phylogenetic analysis considering the MP coding regions of the GPGV variants discussed in this manuscript. The alignment was prepared using the MUSCLE algorithm in Geneious Prime (version 2024.0.7). The tree was constructed using Geneious Tree Builder with the Jukes-Cantor model and the Neighbour-Joining method using 1000 bootstrap replicates. Light blue indicates the asymptomatic, while dark blue indicates the symptomatic clade.

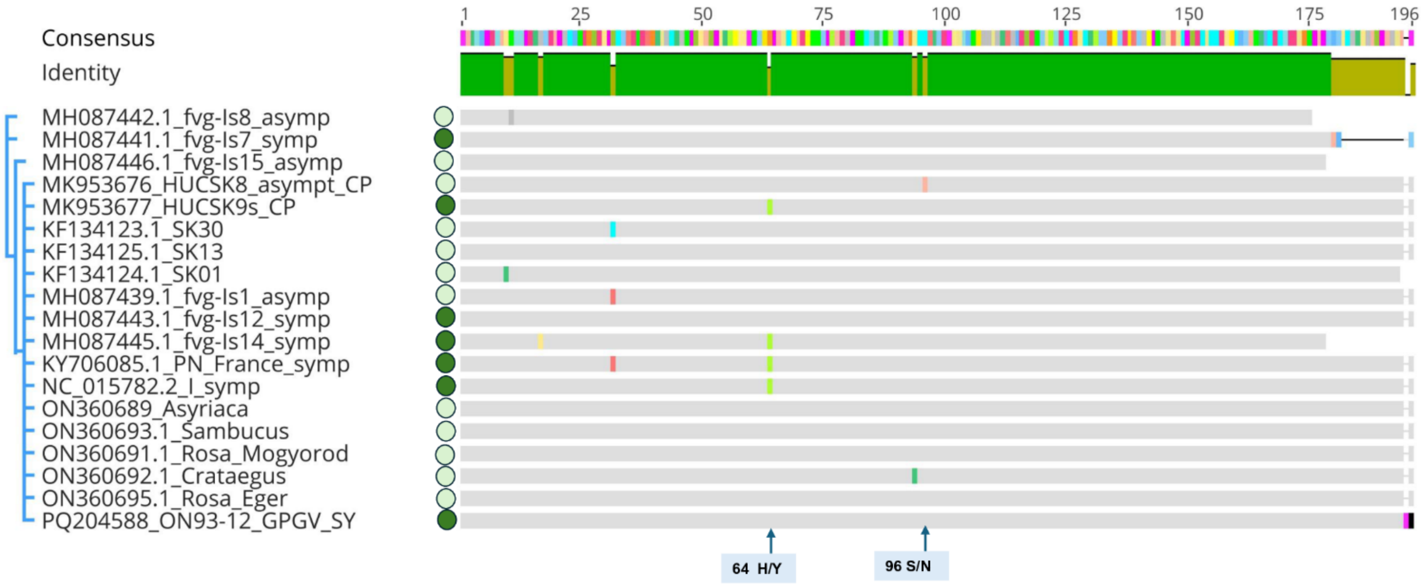

Figure S4.

Multiple alignment of the amino acid sequences of the CP of the different GPGV variants. The alignment of amino acid sequences of GPGV-CP encoded by ORF3 was prepared using the MUSCLE algorithm in Geneious Prime (version 2024.0.7). The differences are marked with different colours. Light green circles indicate the asymptomatic, while dark green circles indicate the symptomatic clustering behaviour of the nucleotide sequence of the variants. Arrows indicate the amino acid differences between the HUCSK8as asymptomatic and HUCSK9s symptomatic variants, whose VSR activity was tested later in this study.

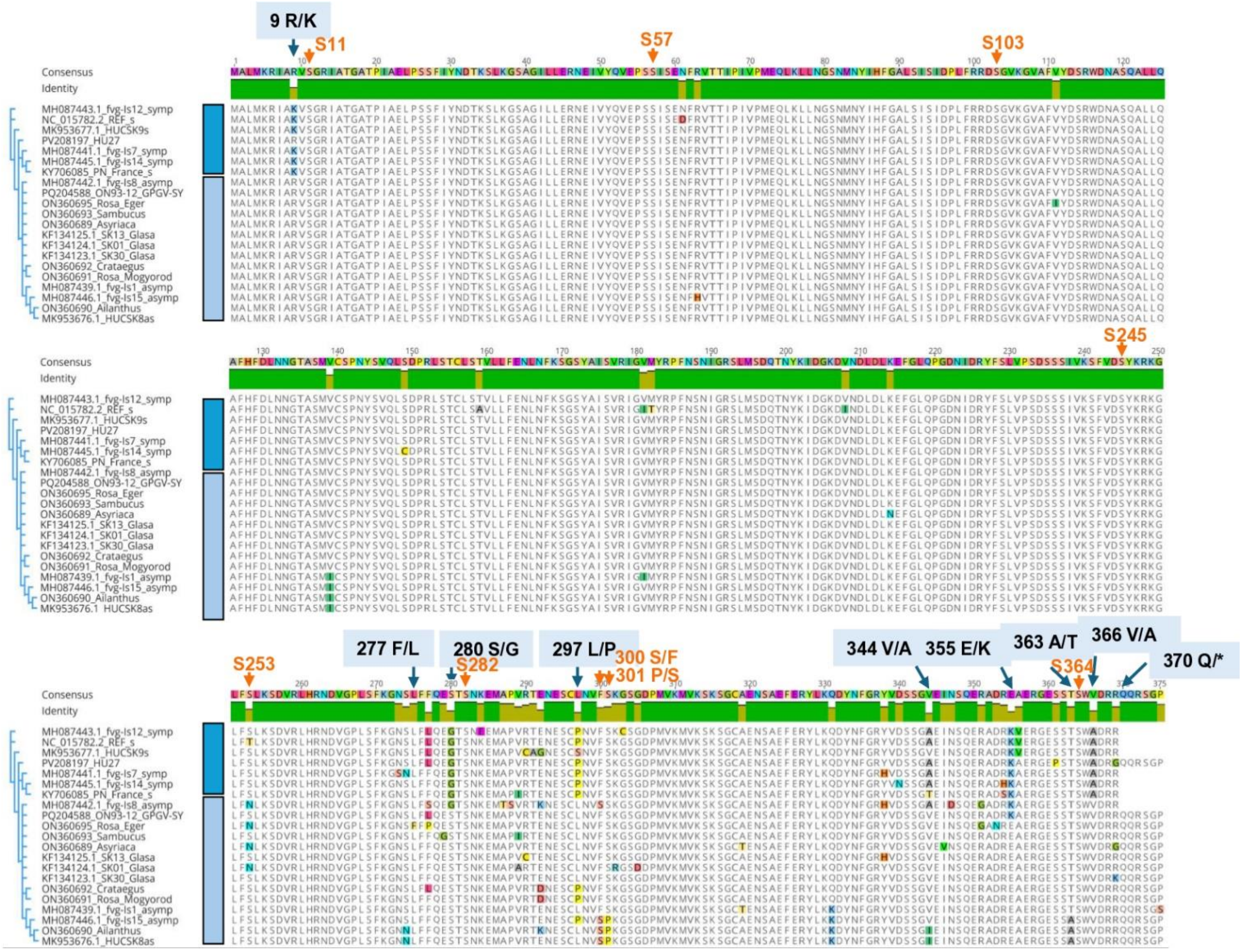

Figure S5. Multiple alignment of the amino acid sequences of the MP of the different GPGV variants. The alignment of amino acid sequences of GPGV-MP encoded by ORF2 was prepared using the MUSCLE algorithm in Geneious Prime (version 2024.0.7). The differences are marked with different colours. Light blue indicates the asymptomatic, while dark blue indicates the symptomatic clustering behaviour of the nucleotide sequence of the variants. Orange numbers indicate the Serine residues, whose phosphorylation was predicted. Numbers in blue boxes indicate the position of the differentiating SNPs, including the amino acid changes.

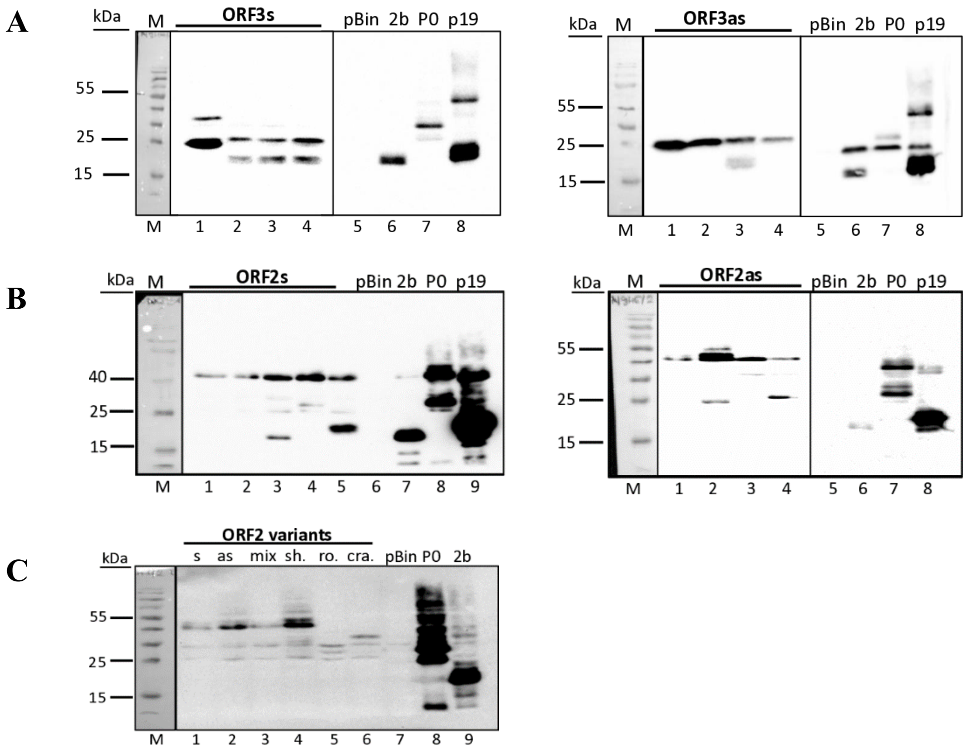

Figure S6.
Testing the protein-coding efficiency of the HA–tagged GPGV-MP and CP expressing constructs using Western blot analyses.
(A) Expression of the CP encoded by the symptomatic and asymptomatic GPGV variants. Western blots were performed using a monoclonal antibody against the HA tag. Lane 1-4: Ha-tagged ORF3s (left panel) and ORF3as (right panel) at the expected sizes of approximately 22 kDa. pBin – empty vector, 2b (12 kDa), P0 (29 kDa), and p19 (19 kDa), M: PageRuler™ Plus Prestained Protein Ladder.
(B) Expression of the MP encoded by the symptomatic and asymptomatic GPGV variants. Lane 1-4: Ha-tagged ORF2s (left panel) and ORF2as (right panel) at the expected sizes of approximately 46 kDa. pBin – empty vector, 2b (12 kDa), P0 (29 kDa), and p19 (19 kDa),
M: ProSieve QuadColor Protein Marker (left panel), PageRuler™ Plus Prestained Protein Ladder (right panel).
(C) Expression of the MP encoded by different natural and recombinant GPGV ORF2s
s (symptomatic), as (asymptomatic), mix (mixed version), sh (short version), ro (rose), cra (crategus). pBin – empty vector, 2b (12 kDa), P0 (29 kDa).

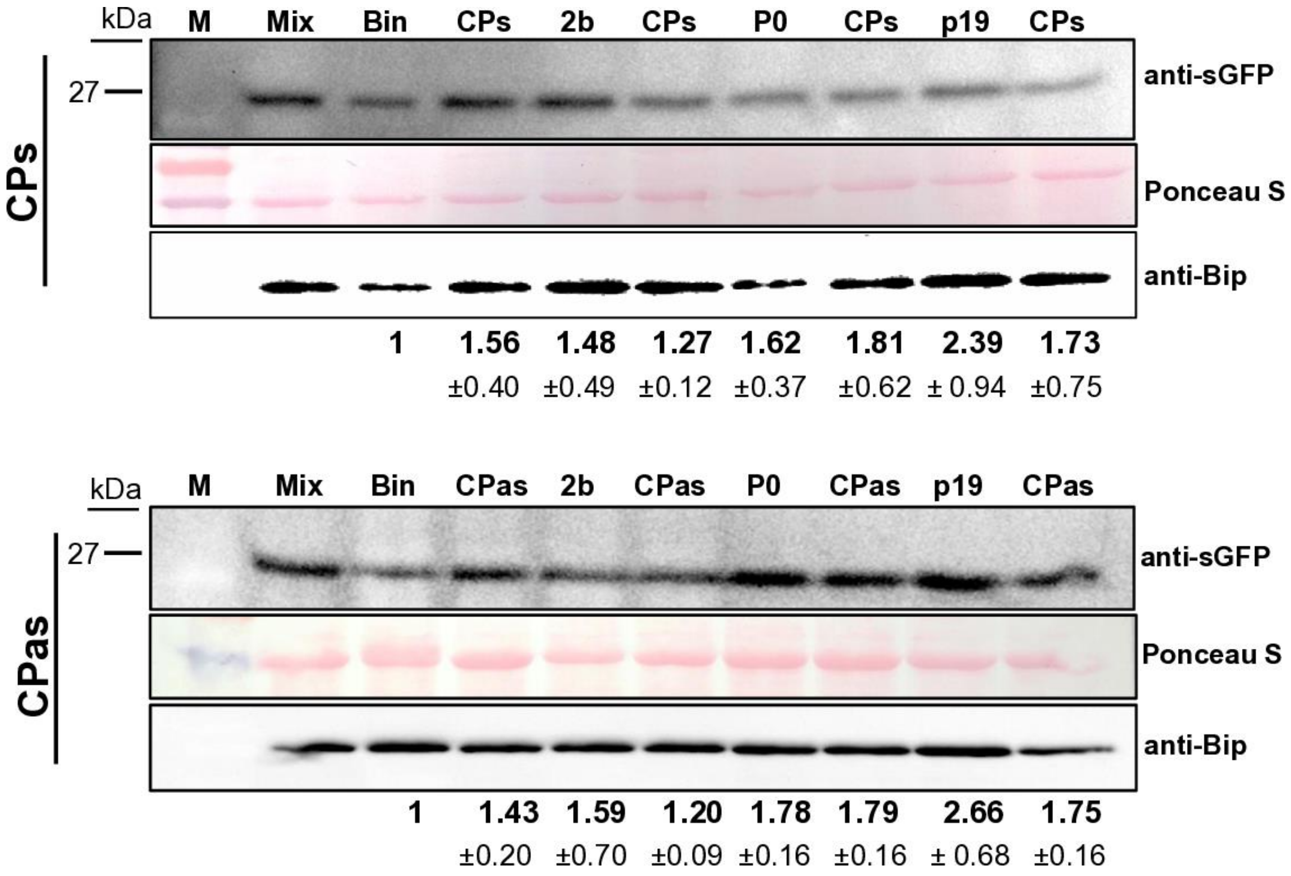

Figure S7.
Western blot analysis of the GFP protein level in the transient assay, when the VSR activity of the GPGV-CP was tested. Photos of the Western blot analysis of one experiment (out of the three biological replicates) are shown here. The upper panel of each block shows the Western blot using GFP antibody. The middle panel shows the membrane stained with Ponceau S, while the lower panel is the Western blot result developed with BiP antibody, which was used as a loading control during the calculation. Each experiment was repeated three times, and the presented numbers refer to the summarised result of the three experiments (± indicates standard deviation, SD).

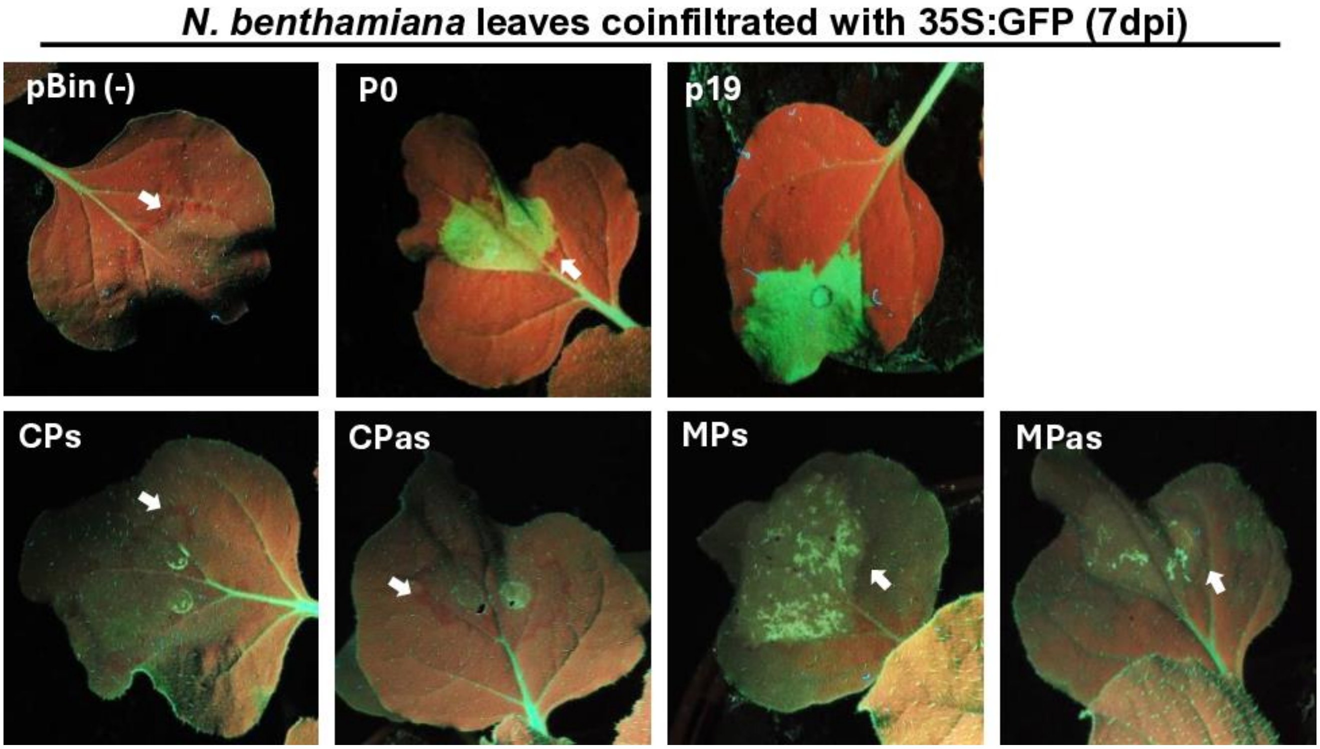

Figure S8.
Transient assay-based test of the systemic VSR activity of the GPGV-CP and GPGV-MP encoded by the symptomatic and asymptomatic variants in the infiltrated leaves. Photographs of the leaves wt. *N. benthamiana* plants at 7dpi after agroinfiltration with GFP and the GPGV-CPs and CPas or GPGV-MPs and MPas constructs under UV light. The empty vector pBin was used as a negative, while $p19^{CymRSV}$ and $P0^{BWYV}$ were used as positive controls. Representative photographs of the upper leaves were taken under long-wavelength UV light at 7 dpi. The development of the “red halo” signal around the agroinfiltrated patches (white arrows) indicated that neither CPs nor CPas nor MPs nor MPas abolishes the signal's movement.

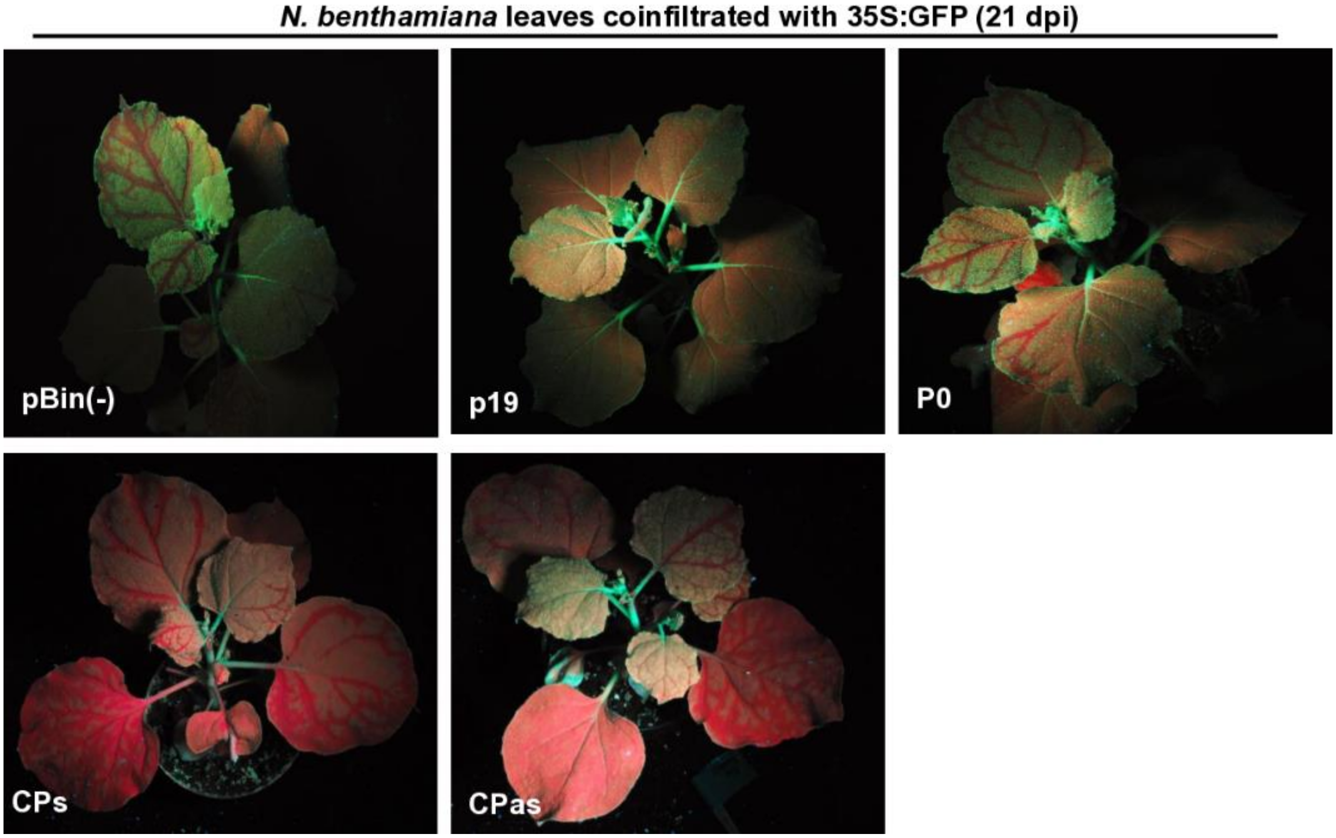

Figure S9.
Transient assay-based test of the systemic VSR activity of the GPGV-CP encoded by the symptomatic and asymptomatic variants in the systemic leaves. Photographs of the leaves of wt. *N. benthamiana* plants at 21 dpi after agroinfiltration with GFP and the GPGV-CP constructs under UV light. The empty vector pBin was used as a negative, while $p19^{CymRSV}$ and $P0^{BWYV}$ were used as positive controls. Representative photographs of the upper leaves were taken under long-wavelength UV light at 21 dpi.

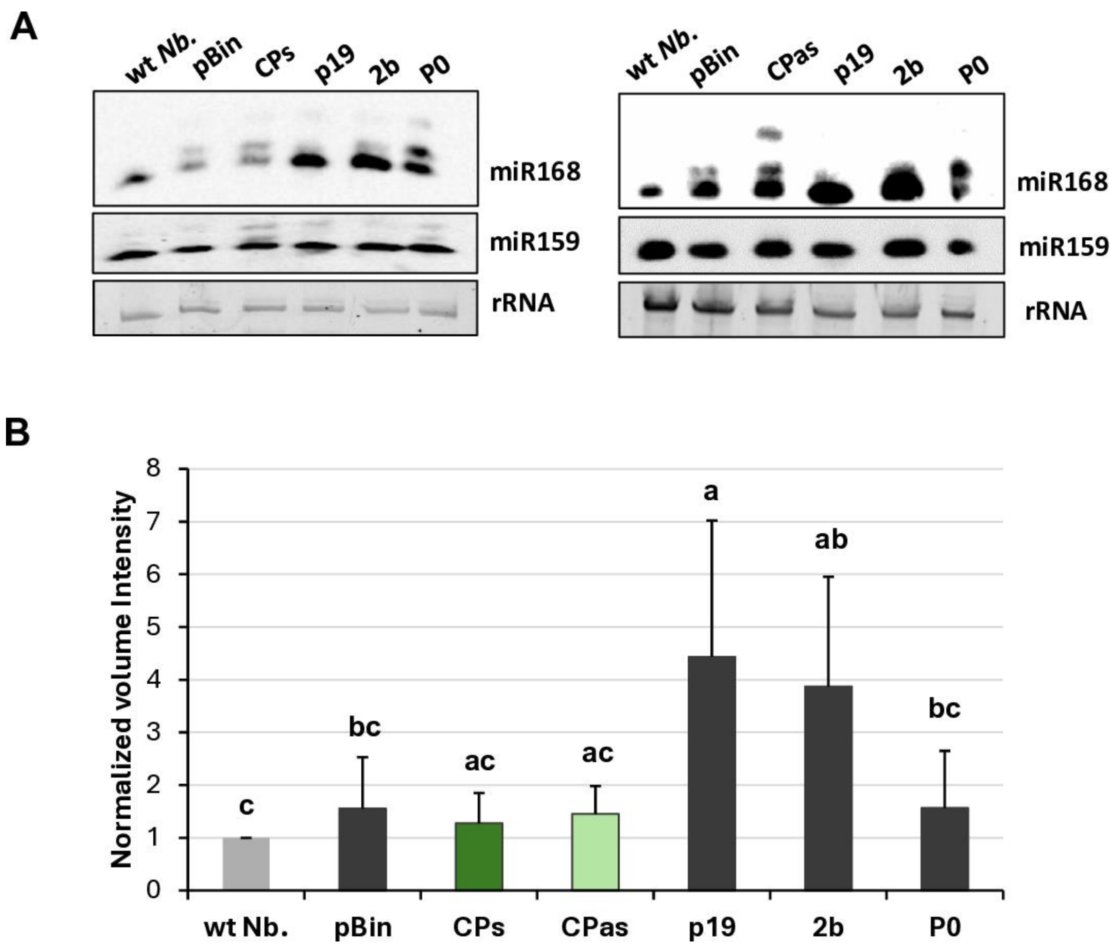

Figure S10.

Analysis of the miR168 expression in the transient assay when the VSR activity of the GPGV-CP was tested.

(A) Result of the miR168-specific Northern blot experiment. miR168 level was monitored using Northern blot analysis and LNA nucleotide-based miR168-specific biotin-labelled probe. The miR168 level of the infiltrated leaves was tested at 4 dpi using Northern blot hybridisation of the siRNAs separated on a polyacrylamide gel. The empty vector pBin was used as a negative, while $p19^{CymRSV}$, $2b^{CMV}$, and $P0^{BWYV}$ were used as positive controls. Non-infiltrated plants (wt. *Nb.*) were also included as negative controls. The ethidium bromide-stained polyacrylamide gel and miR159 LNA probe hybridised membrane were used as a loading control.

(B) Quantification of the miR168 level using a miR168-specific small RNA Northern blot experiment. Mean values were calculated from three independent experiments. The miR168 expression level of the wt *Nb.* was taken as 1. The error bars indicate a standard error, $n = 3$. p19, 2b, and P0 were used as positive controls. Letters indicated a significant difference at the 0.05 level according to one-way ANOVA and Tukey HSD test.

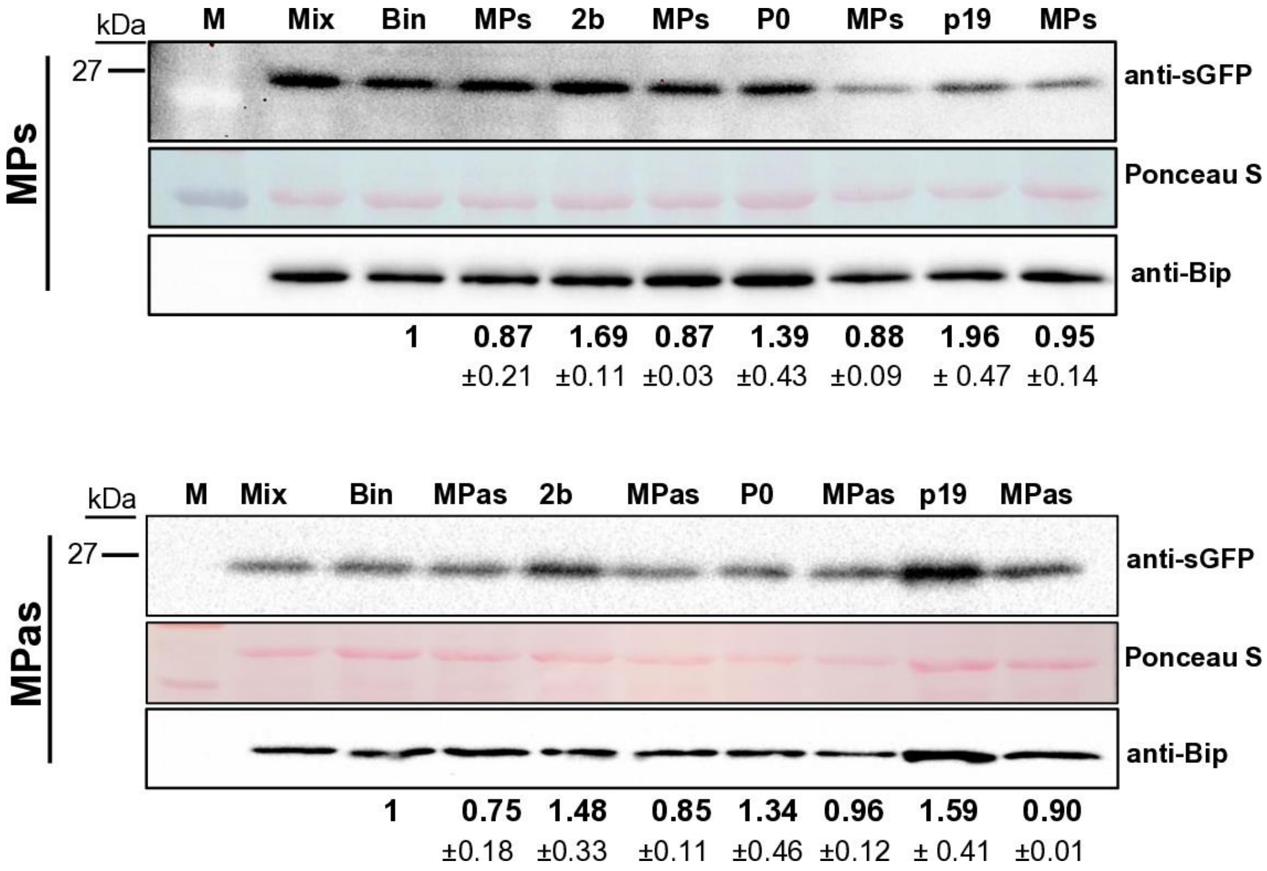

Figure S11.
Western blot analysis of the GFP protein level in the transient assay, when the VSR activity of the GPGV-MP was tested. Photos of the Western blot analysis of one experiment (out of the three biological replicates) are shown here. The upper panel of each block shows the Western blot using GFP antibody. The middle panel shows the membrane stained with Ponceau S, while the lower panel is the Western blot result developed with BiP antibody, which was used as a loading control during the calculation. Each experiment was repeated three times, and the presented numbers refer to the summarised result of the three experiments (± indicates standard deviation, SD).

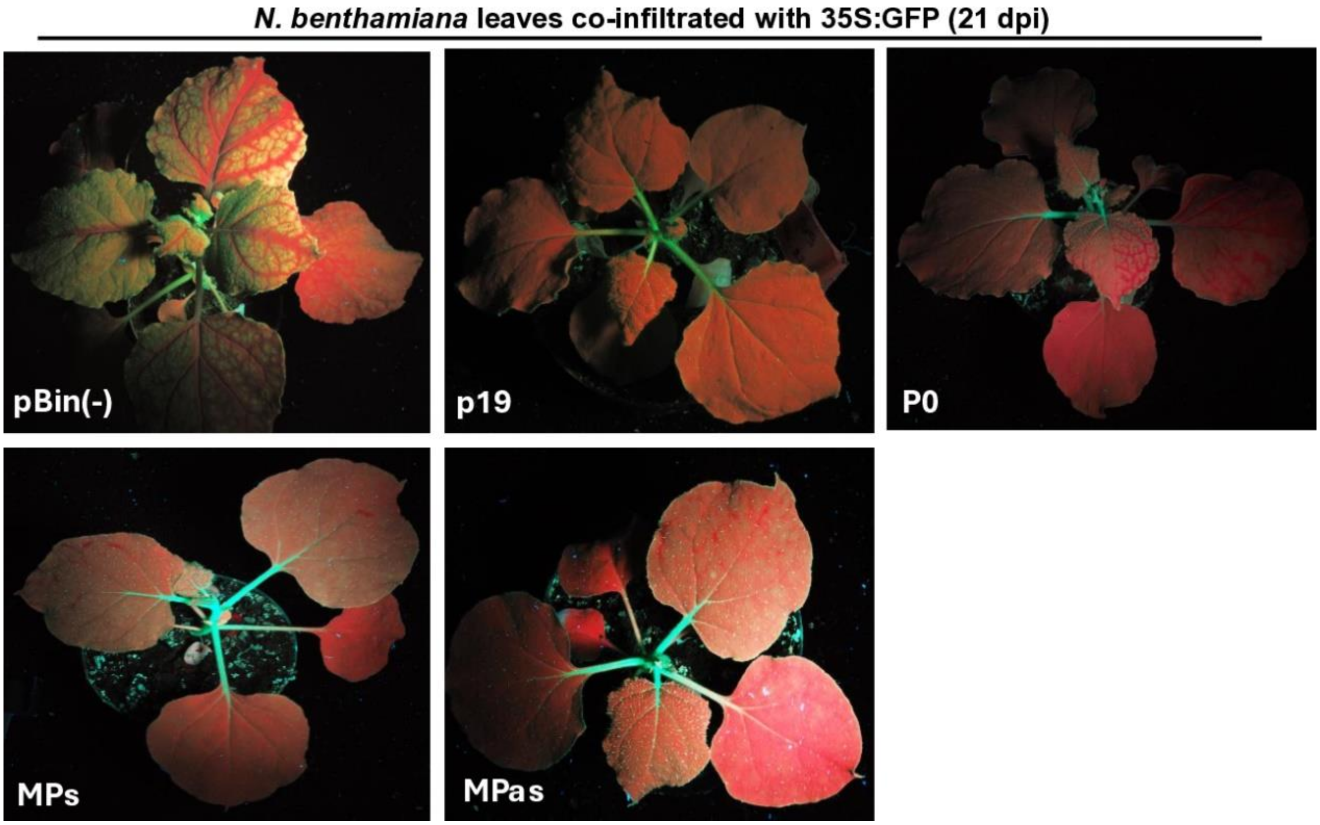

Figure S12.
Transient assay-based test of the systemic VSR activity of the GPGV-MP encoded by the symptomatic and asymptomatic variants in the systemic leaves. Photographs of the leaves of wt. *N. benthamiana* plants at 21 dpi after agroinfiltration with GFP and the GPGV-MP constructs under UV light. The empty vector pBin was used as a negative, while $p19^{CymRSV}$ and $P0^{BWYV}$ were used as positive controls. Representative photographs of the upper leaves were taken under long-wavelength UV light at 21 dpi.

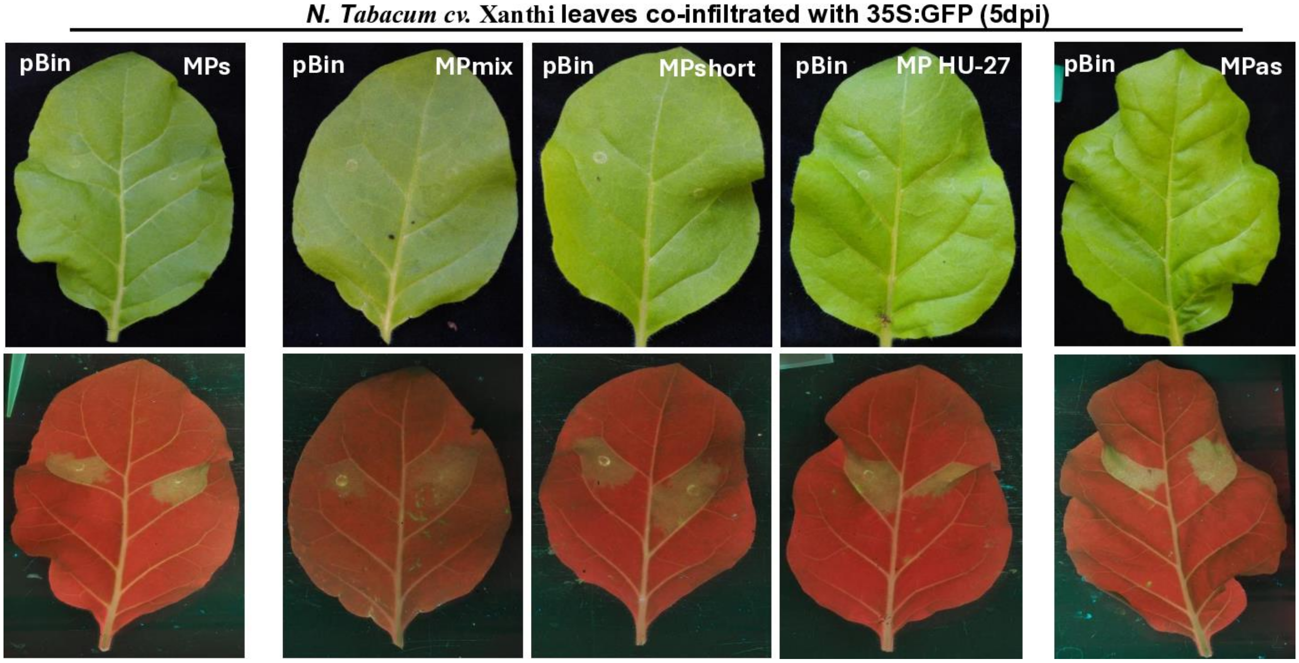

Figure S13.
Transient expression of GPGV-MP variants does not induce necrosis in *Nicotiana Tabacum cv.* Xanthi. Representative photos of agroinfiltration of wt. *Nicotiana Tabacum cv.* Xanthi leaf at 5 dpi after coinfiltration with 35:GFP and GPGV-MPs, MPmix, MPshort, MP HU-27, or MPas at visible light (upper panel) and UV light (lower panel) (n=2). Bold numbers show the quantification of necrotic area measurement using ImageJ software, empty pBin vector as negative control. Each experiment was repeated twice.

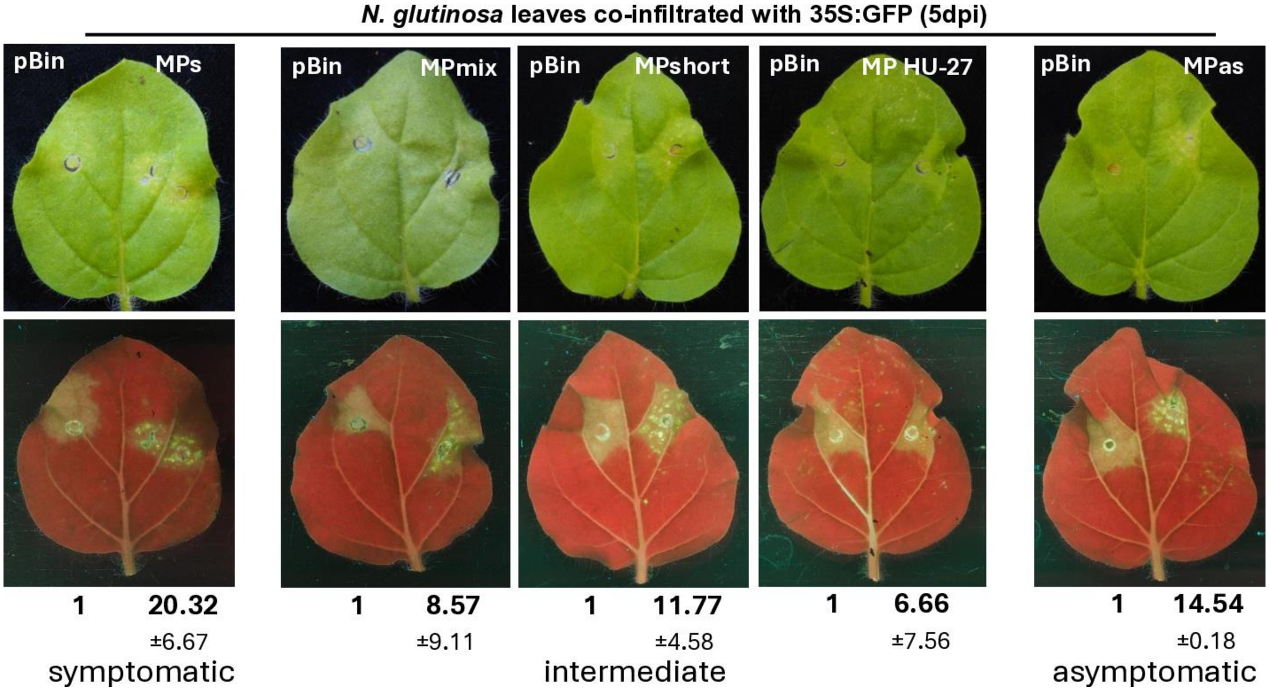

Figure S14. Transient expression of GPGV-MP variants induces mild necrosis in *Nicotiana glutinosa*. Representative photos of agroinfiltration of wt. *N. glutinosa* leaf at 5 dpi after coinfiltration with 35:GFP and GPGV-MPs, MPmix, MPshort, MP HU-27, or MPas at visible light (upper panel) and UV light (lower panel) (n=2). Bold numbers show the quantification of necrotic area measurement using ImageJ software, empty pBin vector as negative control. Each experiment was repeated twice (± indicates standard deviation, SD).

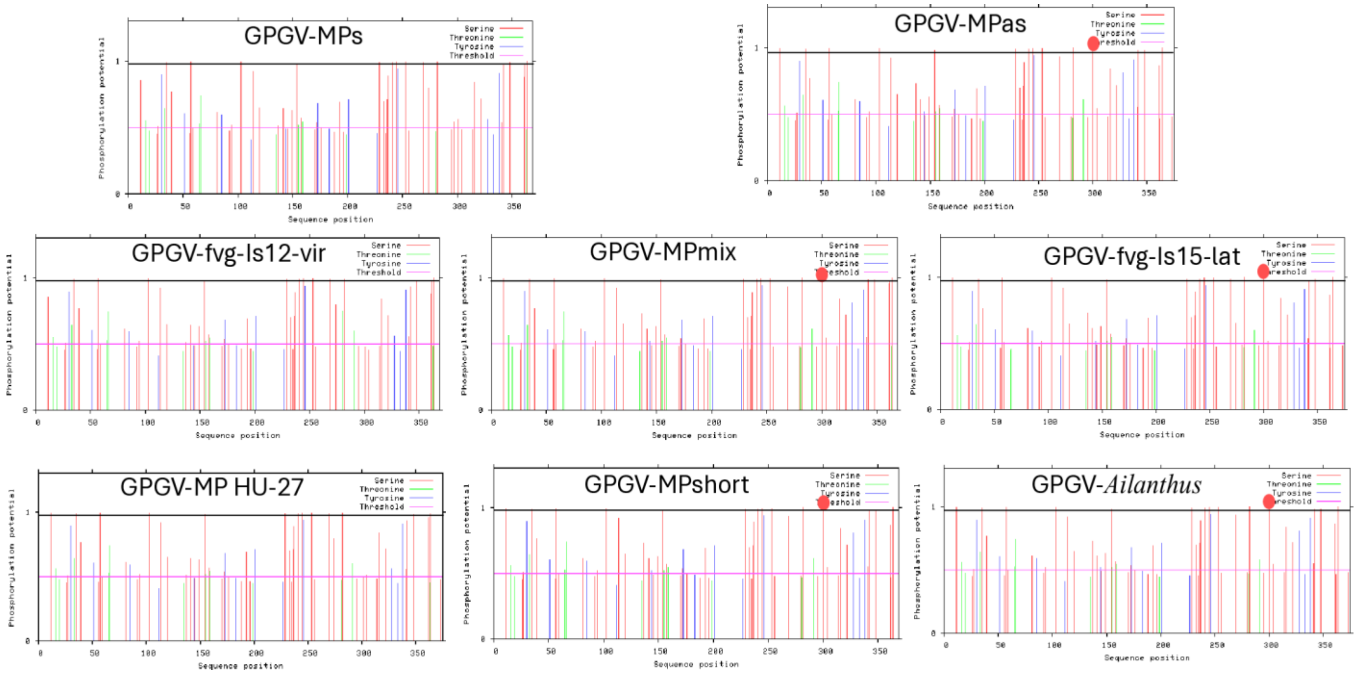

Figure S15.

Prediction of the potential Ser, Tyr, and Thr phosphorylation sites in the GPGV-MP amino acid sequence by NetPhos 3.1. Amino-acid position 300, which is serine only in the asymptomatic variants, is marked with a red sphere. The horizontal violet stripe indicates the threshold value of 0.5 by NetPhos 3.1. The horizontal black line indicates the most likely phosphorylatable sites (score: 0,990).
